# Functional organization of the primary motor cortex in psychosis and the potential role of intereffector regions in psychomotor slowing

**DOI:** 10.1073/pnas.2425388122

**Published:** 2025-10-13

**Authors:** Sebastian Walther, Florian Wüthrich, Anastasia Pavlidou, Niluja Nadesalingam, Stephan Heckers, Melanie G. Nuoffer, Victoria Chapellier, Katharina Stegmayer, Lydia V. Maderthaner, Alexandra Kyrou, Sofie von Känel, Stephanie Lefebvre

**Affiliations:** ^a^University Hospital of Psychiatry and Psychotherapy Bern, Translational Research Center, University of Bern, 3000 Bern, Switzerland; ^b^Translational Imaging Center, Swiss Institute for Translational and Entrepreneurial Medicine, 3000 Bern, Switzerland; ^c^Department of Psychiatry, Psychosomatics, and Psychotherapy, Center of Mental Health, University Hospital of Würzburg, 97080 Würzburg, Germany; ^d^Department of Psychiatry and Behavioral Science, Vanderbilt University, Nashville, TN 37232; ^e^Graduate School for Health Sciences, University of Bern, 3000 Bern, Switzerland; ^f^University Hospital Inselspital Bern, Department for Neurology, Psychosomatic Medicine, 3000 Bern, Switzerland; ^g^Department of Consultation-Liaison Psychiatry and Psychosomatic Medicine, University Hospital Zurich, University of Zurich, 8091 Zurich, Switzerland

**Keywords:** M1, psychomotor slowing, schizophrenia, actigraphy, coin rotation

## Abstract

Altered psychomotor behavior in psychosis is poorly understood. Novel insights into the physiology of the motor cortex prompted a revision of the motor homunculus. Next to core motor effector areas, the primary motor cortex (M1) contains intereffector regions with more integrative connectivity patterns, suggestive of serving psychomotor behavior. Here, we test whether patterns of connectivity differ between patients with and without psychomotor slowing and which cortical areas are associated with aberrant motor behavior in psychosis. This resting-state functional MRI study included 83 patients with psychosis and psychomotor slowing, 43 patients without psychomotor slowing, and 63 matched healthy subjects. We tested group differences in connectivity and regressed motor behavior measures with connectivity in patients with psychomotor slowing. Across subjects, we found distinct rs-fMRI connectivity profiles of the intereffector areas in M1, extending to premotor cortices and cerebellum. Patients with psychomotor slowing had stronger connectivity from the intereffectors than controls or patients without slowing. Finally, motor behavior correlated with connectivity from M1 intereffector regions in patients. Connectivity profiles of intereffectors suggest a role in planning and controlling complex behavioral repertoires. Collectively, these findings in patients with psychomotor slowing stress the importance of intereffector regions in shaping psychomotor behaviors in mental disorders.

## Significance

Recent literature recommended a revision of the human motor homunculus to include, in addition to the primary motor cortex regions active during movement execution, intereffector regions orchestrating complex movement patterns. If intereffectors existed, they would have a key role in shaping movements, potentially with key contributions to peculiar motor behaviors in patients with psychiatric disorders. In a resting-state fMRI dataset, we confirmed the revised motor homunculus organization. Moreover, our data suggest intereffector regions to have distinct functional connectivity patterns in patients with massive psychomotor problems. Intereffector connectivity was linked to measurable psychomotor behaviors. These intereffector regions seem to be key components in the neural circuitry that gives rise to psychomotor abnormalities in psychiatric patients.

Abnormal psychomotor behavior is a transdiagnostic feature of multiple psychiatric disorders (1). Alterations may range from subtle coordination deficits or generalized slowing of movements to a massive disturbance of most nonverbal expressions, such as hand gestures, facial expressions, or body postures. Abnormal psychomotor behavior is associated with poor outcomes and functioning (2, 3). The Research Domain Criteria (RDoC) framework has conceptualized these behavioral abnormalities in the sensorimotor domain of psychopathology (4–7).

Since the early description of psychoses, scholars have speculated about the pathobiology of psychomotor processes (8, 9). For example, Wernicke suspected that internal mind states, movement plans, and ideas would modulate the motor output from the primary motor cortex (M1). However, he could not localize these psychomotor processes. The current understanding of the psychomotor processes in the brain is still limited. Within the RDoC sensorimotor domain, three potential circuits have been discussed: the cortico-subcortical circuit, including basal ganglia and M1 for excitation or inhibition of movements, the cerebello-thalamo-motor circuit for dynamic modulation of movements, and the cortico-cortical circuit including multiple premotor and motor cortical regions, which is thought to orchestrate psychomotor phenomena or movement speed (6). The interplay between these brain regions in the generation of abnormal psychomotor behavior, however, remains unexplained. Northoff and colleagues have argued that bottom–up information flow from basal ganglia, orbitofrontal cortex, or limbic areas to M1 modulates a multitude of psychomotor phenomena, including psychomotor slowing (10). They suggested the balance of resting state functional connectivity (rs-FC) from the raphe nucleus or substantia nigra to M1 and the default mode network would differ between patients with psychomotor agitation vs. retardation. But direct evidence from imaging studies remains scarce.

Few studies have applied neuroimaging in patients with abnormal psychomotor behavior. Structural and perfusion MRI in psychosis patients with motor abnormalities indicated increased neural activity and higher structural connectivity of the premotor cortex, particularly the supplementary motor area (SMA) (11–13). In addition, rs-FC analyses suggested that increased thalamocortical connectivity to M1 was linked to psychomotor phenomena, such as psychomotor slowing or catatonia (14, 15). Likewise, increased thalamocortical connectivity to SMA and M1 was reported in patients with depression and psychomotor retardation (16). In depression, resting perfusion within SMA correlated with reduced motor activity (17, 18). Finally, some studies found altered white matter of motor pathways in patients with psychomotor abnormalities (13, 19–23). Collectively, these data suggest that SMA and M1 along with connected brain areas within the cerebral motor system would shape psychomotor behavior, while the contribution of other potential inputs, e.g., limbic areas or brain regions involved in interoception or motivation, remains poorly understood.

The role of the primary motor cortex in action planning and control has recently been challenged. A large investigation using thousands of rs-FC datasets and other imaging modalities identified classical somatotopic key regions for foot, hand, and mouth that have circumscribed rs-FC to their contralateral homologs as well as to the corresponding areas in the primary sensory cortex (24). However, between these areas, researchers detected so-called Intereffector (IE) regions with a different pattern of rs-FC, suggested to form a somato-cognitive action network (SCAN) (24). Specifically, these Intereffector regions demonstrate strong rs-FC to SMA and cerebellum, critical components of the action-mode network (AMN), suggesting a key role of IE regions in motor planning and motor control (25). Data from microdissections of postmortem brains and intraoperative direct electrical stimulation seem to support the existence of intereffector regions (26). The new model of M1 organization offers a compelling explanation for effective inputs to motor behavior from distant brain areas and aligns with data from nonhuman primates that show a special role of M1 in the formation of complex behavioral repertoires (25, 27), as well as with task-fMRI data in humans during action planning (28). This novel model of M1 organization will change the way we think about psychomotor processes in mental illness (9). The IE regions are most likely involved in abnormal psychomotor behaviors, given their role and rs-FC to cerebellum and SMA, both sharing critically altered connectivity in psychosis (14, 15, 29, 30).

The current study aimed to test whether the newly detected M1 intereffector regions may be related to psychomotor abnormalities in psychosis. To this end, we probed rs-FC in a large group of patients with psychosis and predominant psychomotor slowing and compared the connectivity patterns to subjects with psychosis without psychomotor slowing and healthy controls. Furthermore, we tested whether rs-FC from the M1 intereffector regions would be associated with motor behaviors in psychosis. As a control experiment, we compared groups on the effector rs-FC (foot, hand, mouth) and their association with behavior as well. We hypothesized that patients with psychomotor slowing would show altered rs-FC from the intereffector regions (categorical approach). Moreover, we expected that this connectivity to be associated with motor behaviors in patients, e.g., physical activity levels (dimensional approach).

## 1. Results

### 1.1. Demographics and Clinical Data.

The three groups did not differ in age or sex (Table 1). Patients with and without psychomotor slowing showed no difference in the dosage of current medication (OLZ), number of episodes, or disease duration. However, patients with psychomotor slowing had higher ratings than nonslowed patients on the Positive and Negative Syndrome Scale (PANSS total, positive, and negative), Salpêtrière Retardation Rating Scale (SRRS), Bush Francis Catatonia Rating Scale (BFCRS), and Unified Parkinson’s Disease Rating Scale Part III (UPDRS). Behavioral assessments revealed that patients with psychomotor slowing had lower manual dexterity and reduced activity levels compared to patients without slowing.

**Table 1.** Demographic and clinical information

Group	HC (n = 63)	PS (n = 83)	Non-PS (n = 43)	Statistics
Women (%)	49%	46%	52%	X2 = 0.5, *P* = 0.776
Age (years)	37.6 ± 12.2	36.4 ± 12.2	37.3 ± 12.1	F = 0.2, *P* = 0.820
Education (years)	16.3 ± 2.7	13.1 ± 2.2	14 ± 2.9	F = 14.9, p < 0.001
OLZ equivalents (mg/d)	/	16.6 ± 11.6	14.3 ± 10.4	F = 1.3, *P* = 0.262
Episodes (n)	/	4.6 ± 4.2	5.5 ± 5.5	F = 1.2, *P* = 0.290
Duration of illness (years)	/	10.3 ± 10.4	11.7 ± 10.8	F = 0.5, *P* = 0.471
PANSS total	/	80.3 ± 17.5	57.7 ± 15.2	F = 54.1, *P* < 0.001
PANSS negative	/	24 ± 5.9	14.3 ± 4.61	F = 92.9, *P* < 0.001
PANSS positive	/	15.7 ± 5.3	13.4 ± 4.7	F = 5.8, *P* = 0.018
SRRS	0.5 ± 0.9	24.1 ± 6.3	8.6 ± 11.3	F = 527.8, *P* < 0.001
				HC-PS t = −26.4, *P* < 0.001
				HC- non-PS t = −7.84, *P* < 0.001
BFCRS	0.1 ± 0.4	5.7 ± 4.3	1.5 ± 2.2	F = 63.8, *P* < 0.001
				HC-PS t = −8.5, *P* < 0.001
				HC- non-PS t = −2.3, *P* = 0.09
				PS-non-PS t = 6.4, *P* < 0.001
UPDRS III	0.6 ± 1.1	20.8 ± 11.0	7.6 ± 6.2	F = 118.0, *P* < 0.001
				HC-PS t = −12.5, *P* < 0.001
				HC- non-PS t = −4.4, *P* < 0.001
				PS-non-PS t = 7.9, *P* < 0.001
CR	13.4 ± 3.3	11.4 ± 3.5	12.2 ± 3.5	F = 15.7, *P* < 0.001
				HC-PS t = 5.54, *P* < 0.001
				HC- non-PS t = 3.36, *P* < 0.01
				PS-non-PS t = −1.38, *P* = 0.38
Activity level (counts/h) (subset)	20763.5 ± 6965.1 (n = 40)	12822.8 ± 4926.1 (n = 76)	20171.1 ± 3.7 (n = 23)	F = 27.7, *P* < 0.001
				HC-PS t = 6.7, *P* < 0.001
				HC- non-PS t = 0.4, *P* = 0.90
				PS-non-PS t = −4.4, *P* < 0.001

Olanzapine equivalents (OLZ), Positive And Negative Syndrome Scale (PANSS), Salpêtrière Retardation Rating Scale (SRRS), Bush Francis Catatonia Rating Scale (BFCRS), Unified Parkinson’s Disease Rating Scale Part III (UPDRS), CR: coin rotation, HC: healthy controls, PS: patients with psychomotor slowing, non-PS: patients without psychomotor slowing. L: Left, R: Right,

### 1.2. Seed-to-voxels rs-FC.

#### 1.2.1. rs-FC within the whole sample (n = 189).

**Fig. 1.**
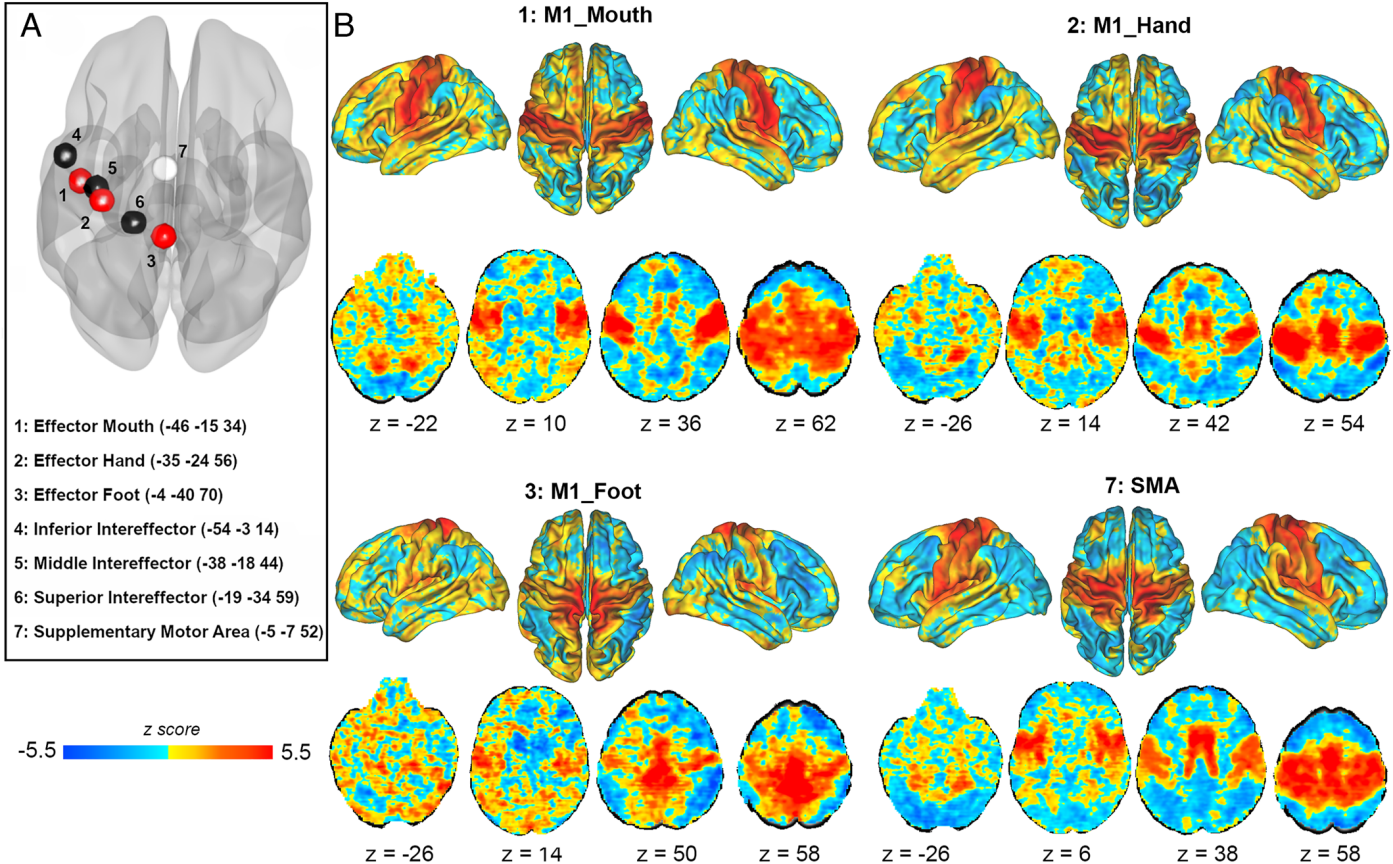
Seed-to-voxels resting-state functional connectivity from M1 effectors and SMA within the whole sample (n = 189). Seed-to-voxels resting-state functional connectivity (rs-FC) from M1 effectors and SMA in the entire sample (combining healthy controls and patient groups). The maps are unthresholded and corrected for age, sex, and framewise displacement as covariates (For thresholded results, see *SI Appendix*, *Appendix A1*). (*A*) Anatomical localization of the seven seeds. The red spheres are the three M1 effectors [1: M1_Mouth (x = −46, y = −15, z = 34), 2: M1_Hand (x = −35, y = −24, z = 56), 3: M1_Foot (x = −4, y = −40, z = 70). The black spheres are the three M1 intereffectors (from *Bottom* to *Top*): 4: M1_Inf_IE (x = −54, y = −3, z = 14)], 5: M1_Mid_IE (x = −38, y = −18, z = 44), 6: M1_Sup_IE (x = −19, y = −34, z = 59), and in white: 7: SMA (x = −5, y = −7, z = 52). (*B*) Seed-to-voxels rs-FC maps from each of the three effectors and the SMA. The color scale reflects positive (yellow-red) and negative (blue) rs-FC between the seeds and the whole brain. Darker areas reflect stronger rs-FC values. Abbreviations: M1_Inf_IE: inferior intereffector, M1_Mid_IE: middle intereffector, M1_Sup_IE: superior intereffector, SMA: supplementary motor area, M1: primary motor cortex, z: axial MNI coordinate.

**Fig. 2.**
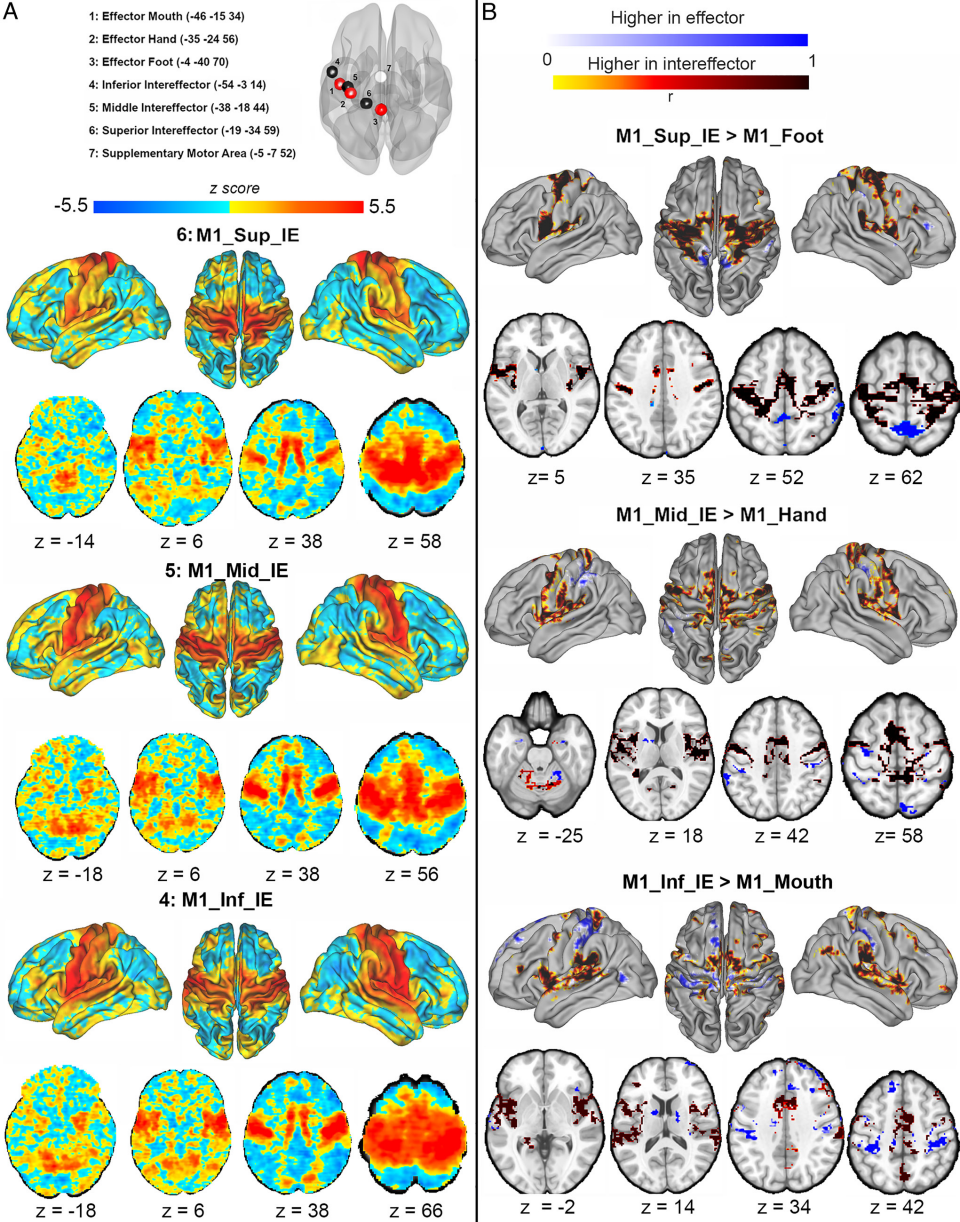
Seed-to-voxels (intereffectors as seeds)resting-state functional connectivity from the intereffectors within the whole sample (n = 189). (*A*) Seed-to-voxels rs-FC from the three intereffectors [M1_Inf_IE (x = −54, y = −3, z = 14)], M1_Mid_IE (x = −38, y = −18, z = 44), [M1_Sup_IE (x = −19, y = −34, z = 59)] in the entire sample (combining healthy controls and patient groups). The color scale reflects positive (yellow-red) and negative (blue) connectivity between the seed and the whole brain. Darker areas reflect stronger rs-FC values. (*B*) Comparisons of the rs-FC between intereffectors and adjacent effectors. Blue scale indicates higher connectivity with the effector seeds while yellow-brown scale indicates higher connectivity with the intereffector seeds. Darker areas reflect stronger rs-FC. Abbreviations: M1_Inf_IE: inferior intereffector, M1_Mid_IE: middle intereffector, M1_Sup_IE: superior intereffector, SMA: supplementary motor area, M1: primary motor cortex, z: axial MNI coordinate.

The anatomical location of the seven seeds, including the three M1 effectors (M1_Foot, M1_Hand, M1_mouth), the three intereffectors (IE) (inferior, M1_Inf_IE, middle, M1_Mid_IE, and superior, M1_Sup_IE), and the SMA are given in Fig. 1*A*. The effector-specific rs-FC maps (Fig. 1*B* and *SI Appendix*, *Appendix A*1) included positive correlations with the cerebellum (IV/V-VIII), bilateral M1, primary sensory cortex (S1), ventral premotor cortex, and SMA. Furthermore, the M1_Hand and M1_Mouth effectors showed high rs-FC with most sensorimotor and premotor areas, while the M1_Foot effector showed high rs-FC predominantly within the bilateral M1 foot/leg areas. In contrast, the three intereffector rs-FC maps showed positive correlations with insula, and anterior cingulate cortex in addition to the cortico-cerebellar motor network (Fig. 2*A* and *SI Appendix*, *Appendix A*1). The rs-FC map of the SMA was similar to the intereffector regions with extensive cerebellar rs-FC. The complete information and coordinates of the clusters are reported in *SI Appendix*, *Appendix A*1. The comparison between the rs-FC of the anatomically closest effectors and intereffectors highlighted specific differences, suggesting distinct roles of the effector and intereffector regions (Fig. 2*B* and *SI Appendix*, *Appendix A*1). We observed similar results when the analyses were restricted to the healthy controls (*SI Appendix*, *Appendix B*1 *and B*2).

We evaluated the robustness of these results by comparing the results of the whole sample to those of ten subsets, each excluding 10 random participants. For each of the seven seeds, the mean Dice Similarity Coefficient (DSC) across the ten subsets demonstrates excellent robustness (All mean DSC>0.75) (*SI Appendix*, *Appendix C*1).

#### 1.2.2. Between-group comparisons of rs-FC.

**Fig. 3.**
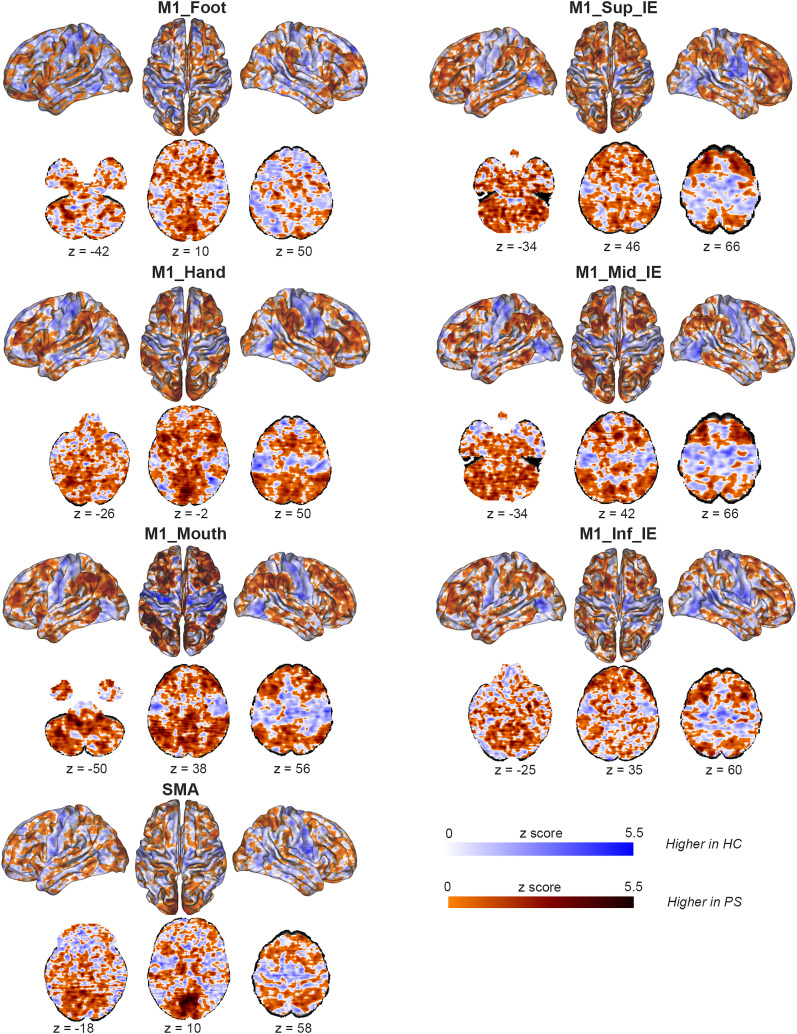
Differences in seed-to-voxels resting-state functional connectivity between patients with psychomotor slowing and healthy controls. The figure displays the unthresholded maps of the seed-to-voxels rs-FC between patients with psychomotor slowing and healthy controls corrected for age, sex, and framewise displacement (For thresholded results, see *SI Appendix*, *Appendix D1*). Each of the three M1 effectors [M1_Foot (x = −4, y = −40, z = 70), M1_Hand (x = −35, y = −24, z = 56), M1_Mouth (x = −46, y = −15, z = 34)], and three M1 intereffectors [M1_Sup_IE (x = −19, y = −34, z = 59), M1_Mid_IE (x = −38, y = −18, z = 44), M1_Inf_IE (x = −54, y = −3, z = 14)]; SMA (x = −5, y = −7, z = 52) were used as seeds. The orange-brown scale reflects higher rs-FC in patients compared to healthy controls, while the blue scale reflects higher rs-FC in healthy controls compared to patients. Darker areas reflect stronger rs-FC. Abbreviations: M1_Inf_IE: inferior intereffector, M1_Mid_IE: middle intereffector, M1_Sup_IE: superior intereffector, SMA: supplementary motor area, M1: primary motor cortex, z: axial MNI coordinate.

**Fig. 4.**
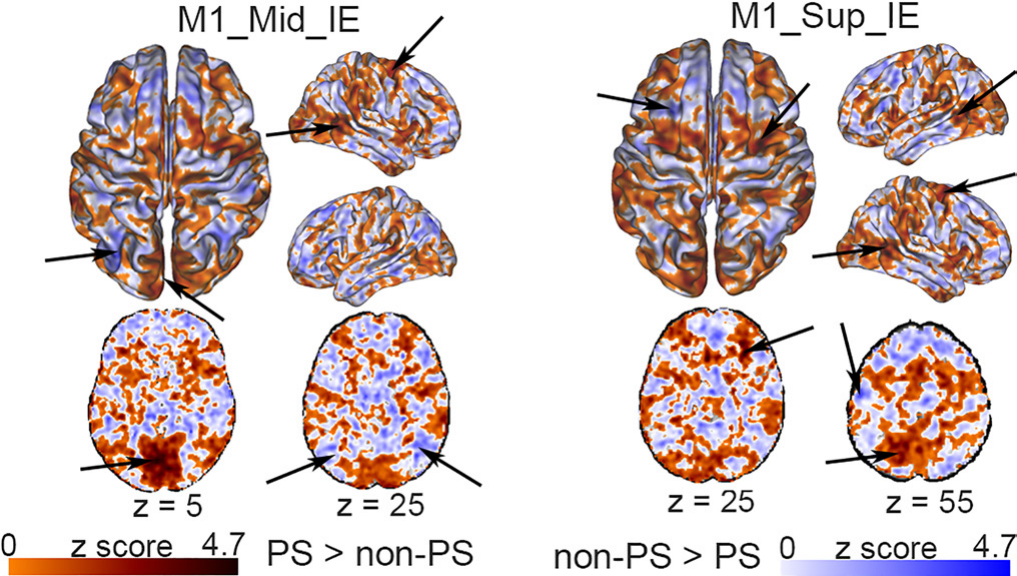
Differences in seed-to-voxels resting-state functional connectivity between patient groups. The figure displays the unthresholded maps of seed-to-voxels rs-FC between the two patient groups corrected for age, sex, framewise displacement, medication dosage, and severity of the disorder using PANSS total score (For thresholded results, see *SI Appendix*, *Appendix D1*). Only the rs-FC with M1_Mid_IE (x = −38, y = −18, z = 44) and M1_Sup_IE (x = −19, y = −34, z = 59) as seeds showed between-patient group differences. The orange-brown scale reflects higher rs-FC in patients with psychomotor slowing compared to patients without psychomotor slowing, while blue scale reflects higher rs-FC in patients without psychomotor slowing than in patients with psychomotor slowing. Darker areas reflect stronger rs-FC. The arrows indicate the brain areas where the difference between the groups would survive the thresholding (voxel-level cluster-forming threshold of *P* < 0.005, followed by a cluster-size threshold of p_(FDR)_ < 0.05). Abbreviations: M1_Mid_IE: middle intereffector, M1_Sup_IE: superior intereffector, PS: patients with psychomotor slowing, non-PS: patients without psychomotor slowing, z: axial MNI coordinate.

We found striking differences between groups in seed-to-voxels rs-FC. Healthy controls and patients with psychomotor slowing differed in rs-FC maps for all seeds (Fig. 3 and *SI Appendix*, *Appendix D*1). Patients with psychomotor slowing had higher rs-FC than healthy controls between M1 effectors, bilateral cerebellum (IV-V-VI), parietal, and prefrontal cortices, most pronounced for the M1_Hand effector. Patients with psychomotor slowing also had higher rs-FC than healthy controls from the intereffectors and SMA to bilateral cerebellum (IV-V-VI- VIII), pulvinar, centromedian thalamus, and prefrontal cortex. At the M1_Hand effector, patients with psychomotor slowing had lower rs-FC than healthy controls to bilateral M1, S1, and premotor cortex (Fig. 3 and *SI Appendix*, *Appendix D*1). Finally, differences between healthy controls and patients without psychomotor slowing (*SI Appendix*, *Appendix D*1 *and D*2) were less pronounced and located at the sensorimotor cortex. Likewise, patients with psychomotor slowing (Fig. 4 and *SI Appendix*, *Appendix D*1) had higher rs-FC than patients without psychomotor slowing from M1_Sup_IE and M1_Mid_IE to parieto-occipital cortices and left pre/postcentral gyrus. Patients with psychomotor slowing had lower rs-FC than patients without slowing from M1_Mid_IE to the right frontal eye field and parietal cortex. However, patient groups lacked differences for the SMA or M1 effectors.

We evaluated the robustness of the significant differences between patient groups by comparing them with 20 subsets, each excluding two random participants. For each of the seven seeds, the mean dice similarity coefficient (DSC) across the 20 subsets demonstrates excellent robustness (All DSC > 0.85) (*SI Appendix*, *Appendix C*1).

#### 1.2.3. Association of effector and intereffector connectivity and motor behavior.

##### 1.2.3.1. Objective physical activity.

**Fig. 5.**
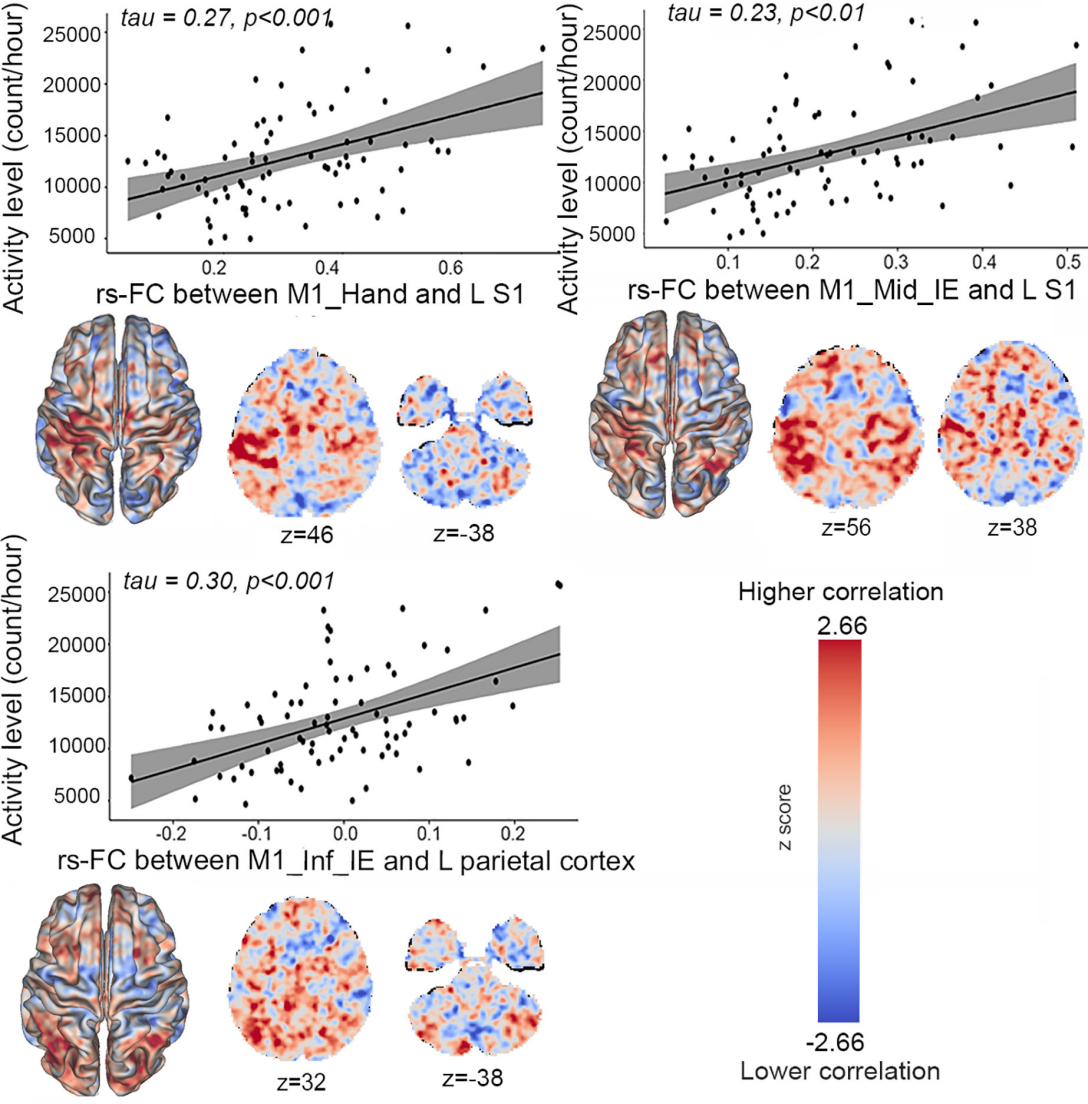
Association between seed-to-voxels resting-state functional connectivity and activity level in patients with psychomotor slowing. This figure displays the association of activity level and seed-to-voxels rs-FC. Rs-FC between M1_Hand [seed (x = −35, y = −24, z = 56)] and left S1, M1_Mid_IE [seed (x = −38, y = −18, z = 44)] and left S1, and M1_Inf_IE [seed (x = −54, y = −3, z = 14)] and left parietal cortex show positive associations with the activity levels in patients with psychomotor slowing. The rs-FC maps are unthresholded and corrected for age, sex, framewise displacement, medication dosage, and the severity of the disease using PANSS total score (For thresholded results see *SI Appendix*, *Appendix E1*). The red scale reflects positive correlations between the rs-FC and behavioral assessment while the blue scale reflects negative correlations. In the scatter plots, the solid line is the line of best fit. The gray shading around the black line represents the 95% CI. The correlation coefficients (tau value) and the associated p-values are reported on each scatter plot Abbreviations: M1_Inf_IE: inferior intereffector, M1_Mid_IE: middle intereffector, M1: primary motor area, S1: somatosensory area. R: right, L: left, resting-state functional connectivity (rs-FC), z: axial MNI coordinate.

The three groups demonstrated distinct associations between rs-FC and activity levels from actigraphy. In patients with psychomotor slowing, higher activity levels were associated with higher rs-FC between the M1_Hand and the left postcentral and supramarginal gyrus, between the M1_Inf_IE and the left parietal cortex as well as between the M1_Mid_IE and the left pre/postcentral gyrus (Fig. 5). In patients without slowing, higher activity levels were associated with lower rs-FC between the M1_Mouth and the right precentral gyrus and the left cerebellum (CRUS II) as well as with lower rs-FC between the M1_Sup_IE and the left prefrontal cortex. Finally, in healthy controls, higher activity levels were associated with higher rs-FC between the M1_Foot and the right parieto-occipital cortex. (*SI Appendix*, *Appendix E*1).

##### 1.2.3.2. Manual dexterity.

**Fig. 6.**
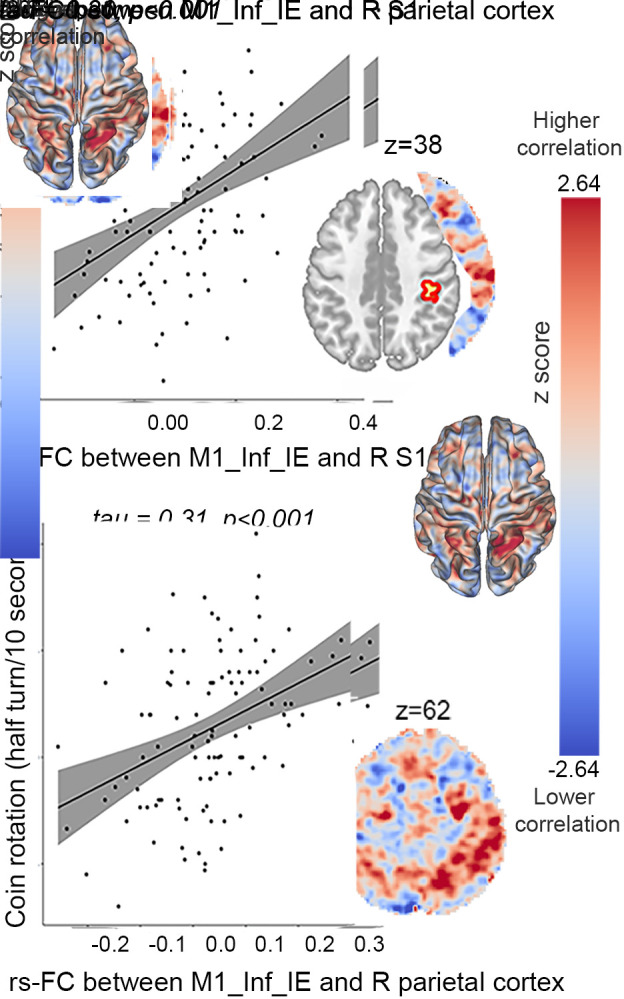
Association between seed-to-voxels resting-state functional connectivity and manual dexterity in patients with psychomotor slowing. This figure displays the significant association of coin rotation score and seed-to-voxels rs-FC. Only the rs-FC between M1_Inf_IE [seed (x = −54, y = −3, z = 14)] and the right somatosensory and parietal cortices show a positive association with the coin rotation score in patients with psychomotor slowing. The rs-FC maps are unthresholded and corrected for age, sex, framewise displacement, medication dosage, and the severity of the disease using PANSS total score (for thresholded results, see *SI Appendix*, *Appendix E2*). The red scale reflects positive correlations between the rs-FC and behavioral assessment while the blue scale reflects negative correlations. In the scatter plots, the solid line is the line of best fit. The gray shading around the black line represents the 95% CI. The correlation coefficients (tau value) and the associated p-values are reported on each scatter plot Abbreviations: M1_Inf_IE: inferior intereffector, M1: primary motor area, S1: somatosensory area. R: right, L: left, resting-state functional connectivity (rs-FC), z: axial MNI coordinate.

The three groups demonstrated distinct associations between rs-FC and manual dexterity. In patients with psychomotor slowing, higher CR scores were associated with higher rs-FC between the M1_Inf_IE and the right parietal cortex and right S1 (Fig. 6). In patients without slowing, CR scores were associated with higher rs-FC between the M1_Hand and the bilateral thalamus. Finally, in healthy controls, there was no association between manual dexterity and the rs-FC between effectors/intereffectors and the rest of the brain (*SI Appendix*, *Appendix E*2).

##### 1.2.3.3. Expert rating of psychomotor slowing in patients with slowing.

**Fig. 7.**
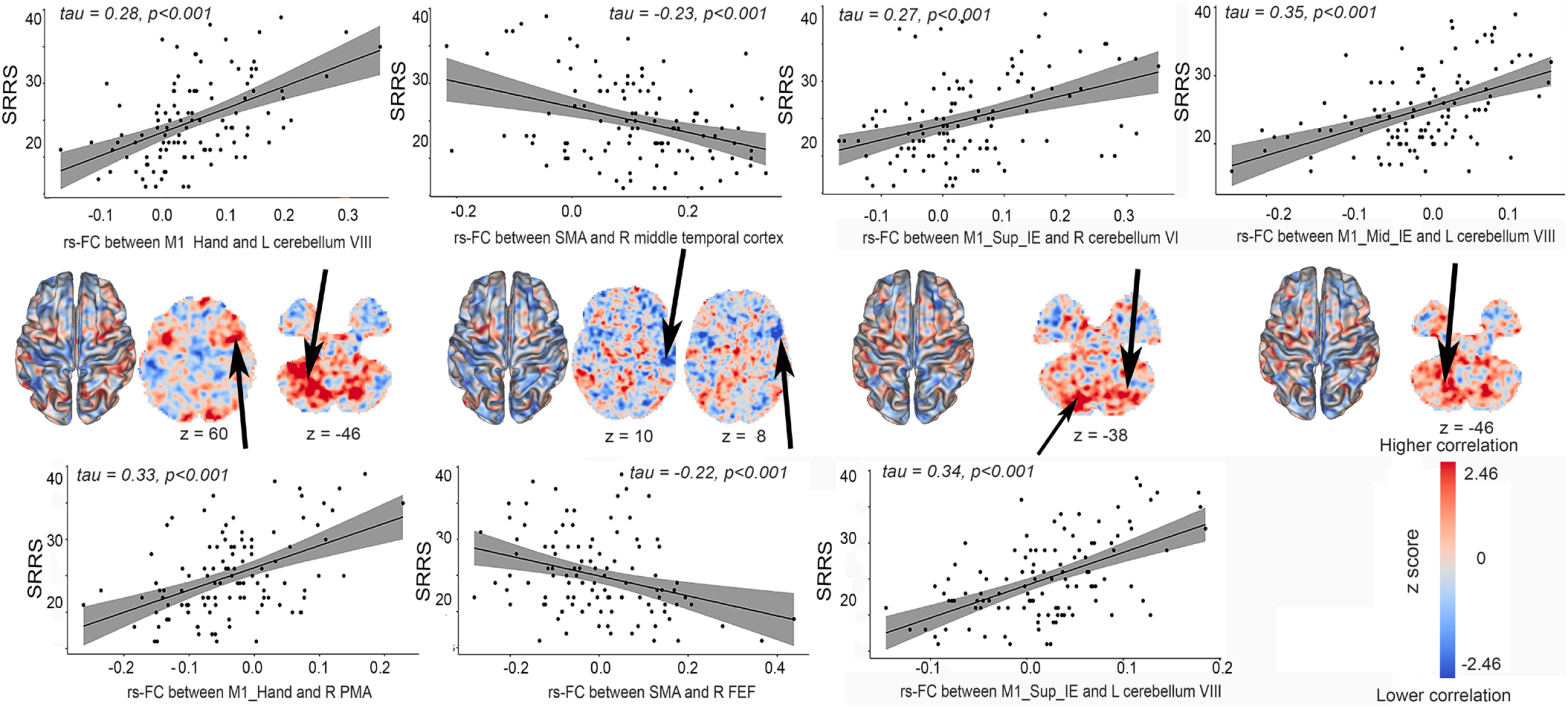
Association between resting-state functional connectivity and the severity of psychomotor slowing in patients with psychomotor slowing. Associations of psychomotor slowing severity as measured by SRRS and rs-FC. The rs-FC maps are unthresholded and corrected for age, sex, framewise displacement, medication dosage, and the severity of the disease using PANSS total score (For thresholded results see *SI Appendix*, *Appendix E3*). The red scale reflects positive correlations between the rs-FC and behavioral assessment while the blue scale reflects negative correlations. Only the significant associations are reported, resulting in M1_Hand (x = −35, y = −24, z = 56), M1_Sup_IE (x = −19, y = −34, z = 59), M1_Mid_IE (x = −38, y = −18, z = 44), and SMA (x = −5, y = −7, z = 52) as seeds. In the scatter plots, the solid line is the line of best fit. The gray shading around the black line represents the 95% CI. The correlation coefficients (tau value) and the associated p-values are reported on each scatter plots The arrows highlight the regions used for the scatter plot. Abbreviations: SRRS: Salpêtrière Retardation Rating Scale, M1_Mid_IE: middle intereffector, M1: primary motor area. M1_Sup_IE: superior intereffector, SMA: supplementary motor area, resting-state functional connectivity (rs-FC), z: axial MNI coordinate.

In patients with psychomotor slowing, higher SRRS scores (more slowing) were associated with higher rs-FC between left M1_Mid_IE and the left cerebellum (VII–VIII), and between left M1_Sup_IE/M1_Hand and the bilateral cerebellum (VII–VIII–IX, CRUS II). More slowing was linked to lower rs-FC from the SMA to the right temporal and bilateral premotor cortex (Fig. 7 and *SI Appendix*, *Appendix E*3). While left M1_Inf_IE rs-FC failed to correlate with slowing severity, more slowing was negatively associated with rs-FC between right M1_Inf_IE and left temporal cortex. Neither the right M1_Sup_IE nor the M1_Mid_IE showed an association with slowing. Finally, as in the left hemisphere, only the rs-FC of M1_Hand effector showed positive associations with slowing: between right M1_hand and left cerebellum VIII or right premotor cortex (*SI Appendix*, *Appendix E*3).

## 2. Discussion

Here, we aimed to test whether the recently discovered organizational pattern in M1 was differentially associated with psychomotor slowing in psychosis. To this end, we investigated rs-FC in the motor circuit in a large sample of patients with psychosis and current psychomotor slowing. Four main findings emerged: 1) In the combined sample as well as in the healthy controls, we corroborated the distinct rs-FC profiles of the intereffector areas in M1 that are similar to those of the SMA (24). 2) Patients with psychomotor slowing had stronger seed-to-voxels rs-FC from the intereffectors than healthy controls or patients without slowing. 3) In patients with psychomotor slowing, motor behavior was correlated with rs-FC from the middle and inferior intereffectors. Thus, the findings suggest a specific role of the M1 intereffector regions of the SCAN in regulating psychomotor behaviors (9, 24). In contrast, rs-FC of the three M1 effector regions (hand, foot, mouth) differed between patients with psychomotor slowing and healthy controls, but only at the mouth effector did we find a difference between healthy controls and patients without slowing. The patient groups lacked any differences in M1 effector rs-FC. Within the patients with psychomotor slowing, only the hand effector displayed a correlation between rs-FC and SRRS scores, while such correlations were seen in superior and middle intereffectors and the SMA.

In line with Gordon et al (24), we found a distinct pattern of rs-FC within M1, segregating the M1 effectors from M1 intereffector regions and the SMA across all subjects. Effectors and their adjacent intereffectors differed in connectivity despite close spatial proximity. Specifically, intereffectors had higher rs-FC to motor and premotor areas, such as SMA, anterior cingulate cortex (ACC, BA32), M1, S1, superior parietal lobule (SPL), putamen, and cerebellum. This pattern of rs-FC to key components of the action mode network suggests task specificity during motor planning and motor control (25).

Our findings are further in line with human neuroimaging work pointing to segregated brain activity and rs-FC within M1 (31). Furthermore, evidence from complex kinematic analyses suggests that M1 hand representations do not follow muscle regions per se, but instead code patterns of activities related to hand use (32). The kinematic features of hand postures are encoded in M1, but also SMA, ventral premotor as well as inferior parietal cortex (33). In addition, task fMRI studies at 7 T reported neural activity in M1 and S1 in identical areas during finger movement planning and execution (28). Finally, the rs-FC pattern from intereffector areas is very similar to neural activity during complex finger movements (34). Animal work suggests effective action maps rather than topography as organizational principle in M1 (35). In nonhuman primates, complex hand movements during reaching or grasping are coded via synergistic neural activity of basic movement patterns, which would allow for simplified movement control (36). Indeed, single M1 corticomotoneuronal cells are tuned by the function of the target muscle (e.g., agonist vs. antagonist), not topography (37). M1 neurons may even directly use active suppression to prioritize specific functions (38). Collectively, these findings from human fMRI and animal studies suggest that M1 organization is not identical within the homunculus, but rather includes multiple nodes, such as the intereffector vs. effector segregation. Intereffectors would be an elegant and effective component of the motor system coding for complex behaviors (9, 27). Indeed, microdissection results and direct electrical stimulation seem to support the existence of intereffectors (26). Moreover, intereffectors were shown to receive inputs from the nucleus ruber, while they are tightly linked to the action mode network, further suggesting a specific role in the modulation of complex behaviors (25, 39). Finally, a large study in patients with Parkinson’s disease found increased connectivity between intereffector regions and basal ganglia, thalamus, and cerebellum (40).

Although this study is the first to test M1 functional organization in subjects with psychomotor slowing, some of the findings align with previous work. Increased rs-FC from thalamus to the motor cortex has been frequently reported in all stages of psychoses, including antipsychotic-naive patients (41–43). Importantly, multiple studies found increased thalamocortical rs-FC to M1 to be linked to psychomotor slowing in psychosis (14, 44, 45). In contrast, increased cerebellar rs-FC to cortical motor areas was linked to higher physical activity or better fine motor performance in psychosis (14, 46). Future studies should probe whether psychomotor slowing correlates with specific connectivity between M1 intereffectors and thalamus or cerebellum.

In the current study, patients with psychomotor slowing had higher rs-FC from M1 intereffectors compared to patients without slowing or healthy controls. In contrast, rs-FC from M1 effector regions differed between patients with psychomotor slowing and healthy controls, but not between the patient groups. Thus, psychomotor slowing was associated with specific rs-FC profiles in psychosis, including M1 connections to SMA and cerebellum, further highlighting the specificity of aberrant rs-FC from the intereffectors in psychomotor slowing. Gordon et al. suspected a specific role of the intereffector regions in the SCAN based on their distinct rs-FC profiles (24). Massive cerebellar and SMA rs-FC suggested that intereffectors would orchestrate movement planning and execution via the action mode network (25). In fact, findings from multiple motor tasks suggest impaired motor control and dexterity in psychosis, supporting a specific alteration in intereffector rs-FC (47–54). The three psychomotor circuits proposed within the RDoC framework are probably all connected to the SCAN via thalamus and cerebellum. Given the massive intracortical connections in the third circuit (cortico-cortical), we would expect this one to have the most associations with SCAN (6).

Higher activity levels correlated with higher rs-FC from M1_Mid_IE to pre- and postcentral gyrus and from the M1_Inf_IE to parieto-occipital cortex, which could be explained by the need for coordination and synchronization in whole-body movements. Better manual dexterity performance correlated with higher rs-FC from the M1_Inf_IE and the right parietal and premotor cortices. The affordances of the coin rotation task include stabilization of multiple joints, integration of proprioceptive feedback, and motor planning (55, 56). Finally, psychomotor slowing severity was associated with higher rs-FC from intereffectors to cerebellar regions.

The aberrant hyperconnectivity in the motor system in patients with psychomotor slowing may arise from impaired inhibitory activity. The excitation/inhibition imbalance has been widely discussed in psychosis and may account for altered rs-FC (51, 57, 58). In fact, when we applied paired-pulse TMS paradigms to this sample, we found that M1 physiology was characterized by both decreased excitability and reduced intracortical inhibition in psychomotor slowing (23). Furthermore, studies reported structural alterations in M1 in patients with psychomotor slowing and other hypokinetic motor abnormalities (59–61). Alternatively, hyperconnectivity may be interpreted as a compensatory response and a signal for system-level plasticity (62). It was proposed that synaptic plasticity dysfunction may drive brain network disruption in schizophrenia (63). Importantly, hyperconnectivity has an impact on metabolic cost and communication efficiency. For now, the link between hyperconnectivity and elevated metabolism in patients with psychomotor slowing is unknown.

Of note, reduced rs-FC from the thalamus to the entire sensorimotor network was detected in some studies in patients with schizophrenia and psychomotor slowing as well as in patients with inhibited depression (64, 65). Consecutively, these authors concluded that more rs-FC in the sensorimotor network would indicate increased psychomotor activity, while lower rs-FC would suggest lower psychomotor activity (10). However, this conclusion is challenged by the current data and past reports in schizophrenia and depression (14, 16, 66). The discrepancy might be related to the rs-FC methods applied, i.e., specific M1 seeds vs. the entire sensorimotor network.

Given the abnormalities within the motor system in psychosis, we expected more rs-FC from M1 to basal ganglia (67, 68). However, only a few associations of M1 with putamen were found. We may speculate that basal ganglia are still involved but receive direct input from thalamus, cerebellum, or premotor cortices, and thus, the rs-FC between M1 and basal ganglia was comparably less pronounced.

Although direct electrical stimulation of the SCAN failed to elicit a motor response, the M1 intereffector regions could be ideal candidates to modulate movement plans or aberrant volition (69). The field should test whether stimulation of the intereffector regions may be useful targets for noninvasive brain stimulation in psychosis with motor symptoms. Up to now, only the SMA or DLPFC have been targeted to improve psychomotor slowing (70–72).

### 2.1. Limitations.

The current findings require discussion in the light of some limitations. First, we focused on ROIs from the paper of Gordon et al. instead of running our own precision functional mapping experiments. Thus, we are relying on prior work but at the same time, we were able to test the replicability of the rs-FC patterns. Second, even though our sample included a substantial number of patients with well-characterized and severe psychomotor abnormalities, larger samples would be beneficial to enhance statistical power. Third, we used data from two randomized controlled trials that had similar but different objectives, introducing heterogeneity of the samples. Furthermore, participants who consent to participate in an RCT represent a subgroup of patients with psychoses, introducing some selection bias. Fourth, resting-state connectivity is only one feature of M1 organization that contributes to complex psychomotor behaviors; myelination or attention-control were not reflected in this study. Finally, the brain-behavioral associations reported here do not imply causality, which would have to be tested using longitudinal assessments and brain stimulation techniques.

### 2.2. Summary.

Intereffector regions have distinct rs-FC profiles that suggest a specific role in motor preparation and motor control (24). Psychosis patients with psychomotor slowing present unique alterations of motor behaviors, including aberrant motor coordination and control with group differences to both healthy subjects and psychosis patients without psychomotor slowing (23, 73). Therefore, we expected alterations in the intereffector rs-FC most likely in the patients with psychomotor slowing (74). In fact, patients with psychomotor slowing differed from healthy controls in rs-FC profiles from M1 effectors, intereffectors, and SMA. Concurrently, patients with psychomotor slowing differed from patients without slowing only in intereffector rs-FC. Thus, intereffector rs-FC seems specifically altered in psychomotor slowing. These group differences were accompanied by correlations between intereffector rs-FC and instrumentally assessed physical activity or clinician-rated psychomotor slowing. Collectively, the current findings corroborate the specific role of intereffector regions and the SCAN for motor control in general and suggest a specific association with psychomotor slowing in psychosis.

## 3. Materials and Methods

Full description of the methods is reported in *SI Appendix*, *Appendix F*.

### 3.1. Participants.

We combined baseline data of 201 right-handed participants from two clinical trials (71, 75). The sample included 85 patients with schizophrenia spectrum disorders and psychomotor slowing according to the Salpêtrière Retardation Rating Scale (76) (SRRS total score ≥ 15), 47 patients with schizophrenia spectrum disorders without psychomotor slowing (SRRS score < 15), and 67 age and gender-matched healthy controls (HC). Twelve subjects had to be excluded for poor data quality, leading to a final sample size of n = 189 (slowed group: n = 83, nonslowed group: n = 43, healthy controls: n = 63) (Table 1). The studies adhered to the Declaration of Helsinki and were approved by the local ethics committee (Kantonale Ethikkommission Bern, Switzerland: 2018-02164 and 2019-00798). Participants received written and oral information on the studies and had sufficient time to decide. We ensured that the participants comprehended the information and received clarification in case of questions. Before any study procedures, participants provided written informed consent. Daily dosage of antipsychotics was calculated as mean olanzapine (OLZ) equivalents (77).

General exclusion criteria were active substance dependence except for nicotine, neurological disorders impacting motor behavior, severe brain injury with consecutive loss of consciousness, and contraindications for MR acquisition, i.e., metallic parts in the body, or pregnancy. Additional exclusion criteria for healthy controls were a history of any psychiatric disorder or any first-degree relative with psychosis.

### 3.2. Clinical and Behavioral Assessment.

Clinical and behavioral assessments have been reported previously (71, 73). Briefly, we used the Structured Clinical Interview for DSM-5-TR (SCID-5) to diagnose schizophrenia, schizoaffective, or schizophreniform disorders, and the Positive And Negative Syndrome Scale (PANSS) (78) to assess the symptom severity. Clinical rating scales of motor abnormalities included the Salpêtrière Retardation Rating Scale [SRRS (76)], the Bush Francis Catatonia Rating Scale (BFCRS) (79), and the Unified Parkinson’s Disease Rating Scale Part III (UPDRS) (80). We calculated activity levels in counts/h during wake periods of the day (81–83) using actigraphy (triaxial-accelerometer Move4, movisens GmbH, Karlsruhe, Germany). Manual dexterity of the dominant hand was assessed using the coin rotation task (CR) (73, 84, 85).

### 3.3. MRI Acquisition and Preprocessing.

T1-weighted structural MRI (176 slices of 1 mm) and BOLD resting-state fMRI (rsfMRI) (TR = 1,000 ms, voxel size 2.5 mm^3^ with 600 volumes) were acquired on a 3 T Prisma MRI whole-body scanner using a 20-channel radio-frequency head coil (Siemens, Germany).

Functional connectivity analyses were performed using CONN (86) 22.a and SPM 12.7771. Data preprocessing followed standard pipelines including motion realignment, coregistration between the functional scans and 3D-T1w scans of each participant, normalization to MNI space, and spatial smoothing with a Gaussian filter of 4 mm. Afterward, we denoised the normalized data (87, 88) including regression of potential confounding effects characterized by white matter, CSF, six motion parameters and their first order derivatives (12 factors), session effects and their first-order derivatives (2 factors), and linear trends (2 factors). We applied a bandpass filter of 0.008 Hz and 0.09 Hz, a linear detrending, and despiking to exclude high-frequency fluctuations while minimizing the influence of physiological, head-motion, and other noise sources that might remain after the denoising step. We used absolute head motion threshold and mean frame-wise displacement (FD) [as defined by Power et al. (89)] greater than 0.5 mm as exclusion criteria.

### 3.4. MRI Analyses.

#### 3.4.1. First-level analysis.

To evaluate the resting-state functional connectivity (rs-FC) associated with the motor control of the dominant side, seven seeds (8 mm^3^ spheres) were defined in the left hemisphere based on Gordon et al. (24) including the three intereffector (IE) areas [inferior, M1_Inf_IE (center -54 -3 14); Middle, M1_Mid_IE (center -38 -18 44), and Superior, M1_Sup_IE (center -19 -34 59)], the three M1 effector areas [M1_Hand (center -35 -24 56), M1_Foot (center -4 -40 70), M1_Mouth (center -46 -15 34), and the SMA (center -5 -7 52)] (Fig. 1*A*).

Seed-based rs-FC maps for each of these seven seeds were estimated for each subject. Rs-FC strength was represented by Fisher-transformed bivariate correlation coefficients from a weighted general linear model (weighted-GLM), estimated separately for each seed area and target voxel, modeling the association between their BOLD signal time series.

#### 3.4.2. Second-level analyses.

First, we explored the seed-to-voxels rs-FC for seven seeds (the three intereffectors, the three M1 effectors (foot, hand, and mouth), and the SMA) within the whole sample. Next, we compared rs-FC patterns between adjacent seeds (M1_Foot vs. M1_Sup_IE, M1_Hand vs. M1_Mid_IE, and M1_Mouth vs. M1_Inf_IE). Third, we compared seed-to-voxels rs-FC for the seven seeds between the three groups (healthy controls, patients with psychomotor slowing, and patients without slowing). Fourth, we tested the association of seed-to-voxels rs-FC with objective measures of motor behavior (activity level in a subset of patients with psychomotor slowing, n = 76, healthy controls, n = 40, patients without slowing, n = 23, and manual dexterity) in each of the three groups. Moreover, we tested rs-FC correlation with SRRS within the slowed group. For all the analyses, we included the mean FD, age, and sex as covariates. In addition, analyses performed within and between patient groups included OLZ and PANSS total score as additional covariates to rule out the effect of daily medication and total symptom severity, including negative symptoms.

#### 3.4.3. Cross-validation analyses.

To assess the robustness of rs-FC for the seven seeds, we employed a leave-out cross-validation approach. Ten subsets (n = 179 each) were generated by excluding 10 random participants from the full sample (n = 189). For each seed, seed-based connectivity masks were computed within each subset and compared to the corresponding connectivity mask derived from the full sample using the Dice Similarity Coefficient (DSC). We conducted an additional cross-validation procedure for the seeds that demonstrated rs-FC differences between patient groups (i.e., the middle and superior intereffector regions). Specifically, twenty subsets were created by excluding two random patients from each, and DSC values were computed.

#### 3.4.4. Statistics.

A separate GLM was estimated for each seed (seed-to-voxels rs-FC). Voxel and cluster-level hypotheses were evaluated using multivariate parametric statistics with random-effects across subjects and sample covariance estimation across multiple measurements. The comparison of the connectivity between adjacent seeds represents within-subject effects, while the second-level analyses correspond to repeated-measures analyses of the selected effects (between seed comparisons within the same sample).

Cluster-level inferences were based on parametric statistics from Gaussian Random Field theory, (90, 91) and the maps were thresholded using a combination of a cluster-forming *P* < 0.001 (whole sample rs-FC) or *P* < 0.005 (between-groups comparison) voxel-level threshold, and, to correct for multiple comparisons, a p-FDR(false discovery rate corrected)<0.05 cluster-size threshold (92, 93)—the same thresholds applied for cross-validation analyses.

## Supplementary Material

Appendix 01 (PDF)

## Data Availability

The raw dataset presented in this article is not readily available because some participants did not consent to data sharing. Requests to access the datasets should be directed to S.L., Lefebvre_S@ukw.de. However, statistical MRI maps for group analyses are publicly available on Neurovault (94).
