# Supplementary material for: Functional organization of the primary motor cortex in psychosis and the potential role of intereffector regions in psychomotor slowing
Source: Proc Natl Acad Sci U S A. 2025 Oct 13;122(42):e2425388122. doi: 10.1073/pnas.2425388122 (PMC12557505; doi:10.1073/pnas.2425388122)
Supplement: Supplementary file 1 — Appendix 01 (PDF) [file pnas.2425388122.sapp.pdf]

SI Appendix to:

Functional organization of the primary motor cortex in psychosis and the potential role of intereffector regions in psychomotor slowing

Sebastian Walther[1,2,3], Florian Wüthrich[1,2], Anastasia Pavlidou[1,2], Niluja Nadesalingam[1], Stephan Heckers[4], Melanie G Nuoffer[1,5], Victoria Chapellier[1], Katharina Stegmayer[1], Lydia V Maderthaner[1,6], Alexandra Kyrou[1,7], Sofie von Känel[1,5], Stephanie Lefebvre[1,2] *

* Corresponding author

Table of contents

SI Appendix A: Complementary data on the seed-to-voxels rs-FC at the whole group level……….2

SI Appendix A1: Cluster statistics related to the whole group seed-to-voxels analysis with M1 effectors and intereffectors………………………………………………………………………………..………2

SI Appendix B: Replication of the seed-to-voxels rs-FC in the healthy controls………………………18

SI Appendix B1: Effectors and intereffectors seed-to-voxels rs-FC in healthy controls……………….…*18*
SI Appendix B2: Cluster statistics related to the seed-to-voxels analysis with M1 effectors and intereffectors within the healthy controls. .... …………………*19*

SI Appendix C: Cross validation……………………………………………………………………..………34
SI Appendix C1: Cross-validation of the seed-to-voxels connectivity………………………..……… 34

SI Appendix D: Complementary data on between-group comparisons of the seed-to-voxels rs-FC with M1 effectors and intereffectors 35

SI Appendix D1: Between-groups difference in effectors and intereffectors seed-to-voxel rs-FC.......... 35
SI Appendix D2: Differences in seed-to-voxels resting-state functional connectivity between patients without psychomotor slowing and healthy controls…………………………………………….…39

SI Appendix E: Complementary data on the effectors and intereffectors seed-to-voxel rs-FC association with motor behavior 40

SI Appendix E1: Cluster statistics related to effectors and intereffectors seed-to-voxels rs-FC association with measured activity level.. 40

SI Appendix E2: Cluster statistics related to effectors and intereffectors seed-to-voxels rs-FC association with measured manual dexterity.. 41

SI Appendix E3: Cluster statistics related to effectors and intereffectors seed-to-voxel rs-FC association with SRRS in patients with psychomotor slowing... 42

SI Appendix F: Complementary Methods 43

SI Appendix F1: Extended Methods sections…………………………..……………………………….43
SI Appendix F2: Between-cohorts clinical comparisons…………………………………………………… 47

SI Appendix A: Complementary data on the seed-to-voxels rs-FC at the whole group level

SI Appendix A1: Cluster statistics related to the whole group seed-to-voxels analysis with M1 effectors and intereffectors

	x	y	z	size	p-FDR	Brain Areas
**M1_Foot**						
**Positive connectivity**	-4	-42	72	5773	<0.001	B M1 (BA 4), S1(BA 1), SPL (BA 7), precuneus, SMA (BA 6), PM (BA 6), ant and PCC (BA 31-32)
	18	-44	-58	129	<0.001	R cerebellum (VIII)
	38	-20	20	69	<0.01	R Insula (BA 13)
	-14	-42	-58	65	<0.01	L cerebellum (VIII-IX)
	22	-30	-26	57	<0.01	R cerebellum (IV-V)
	44	-72	-46	50	<0.01	R cerebellum (CRUS II)
	2	-94	20	44	<0.05	R Occipital cx (BA 18)
	-52	-30	18	34	<0.05	L supramarginal Gyrus (BA 40)
	-32	-20	18	32	<0.05	L Insula (BA 13)
**Negative connectivity**	54	-40	54	342	<0.001	R supramarginal Gyrus (BA 40)
	-8	8	16	94	<0.001	L caudate
	44	18	42	53	<0.01	R FEF (BA 8)
	44	-66	48	31	<0.05	R angular cx(BA 39)
**M1_Hand**						
**Positive connectivity**	-36	-24	56	9669	<0.001	B M1 (BA 4), S1(BA 1), SPL (BA 7), precuneus, SMA (BA 6), PM (BA 6), ACC and PCC (BA 31-32)
	16	-52	-22	348	<0.001	R cerebellum (IV-V-VI)
	40	-12	16	316	<0.001	R S1 (BA1), R supramarginal Gyrus (BA 40), R Insula (BA 13)
	22	-54	-54	274	<0.001	R cerebellum (VIII-IX, vermis VIII)
	30	-88	-36	49	<0.01	R cerebellum (CRUS II)
	-4	50	-2	36	<0.05	B AntPFC(BA 10)
**Negative connectivity**	-60	-40	48	370	<0.001	L supramarginal Gyrus (BA 40)
	52	-40	54	273	<0.001	R supramarginal Gyrus (BA 40), R SPL (BA 7)
	6	-60	68	174	<0.001	precuneous

	18	-10	16	60	<0.01	R thalamus (ventral anterior/lateral nucleus)
	-10	6	8	49	<0.01	L caudate
	36	16	62	40	<0.05	R FEF (BA 8)
	-30	-70	-24	40	<0.05	L cerebellum (VI)
**M1_Mouth**						
**Positive connectivity**	-48	-14	36	10884	<0.001	B M1 (BA 4),S1(BA 1),SPL (BA 7), precuneus, SMA (BA 6), PM (BA 6), ACC and PCC (BA 31-32)
	18	-58	-24	205	<0.001	R cerebellum (VI)
	12	-64	-46	153	<0.001	R cerebellum (VIII)
	-16	-66	-18	148	<0.001	L cerebellum (VI)
	-14	48	34	121	<0.001	L DLPFC 8 (BA 9)
	-4	-4	56	86	<0.001	SMA (BA 6)
	-12	-64	-50	68	<0.01	L cerebellum (VIII)
	-4	12	64	57	<0.01	L PM (BA 6)
**Negative connectivity**	34	50	34	90	<0.001	R DLPFC (BA 9)
	8	36	30	59	<0.01	R FEF (BA 8), R ACC (BA 32)
	16	-8	16	43	<0.05	R thalamus (ventral anterior/lateral nucleus)
	-14	0	14	39	<0.05	L caudate
	12	50	24	30	<0.05	R AntPFC(BA 10)
**SMA**						
**Positive connectivity**	0	-8	54	20641	<0.001	B M1 (BA 4),S1(BA 1),SPL (BA 7), precuneus, SMA (BA 6), PM (BA 6), ACC and PCC (BA 31-32)
	24	-54	-54	376	<0.001	R cerebellum (VIII-IX)
	-28	-50	-54	238	<0.001	L cerebellum (VIII-IX)
	28	-46	-26	203	<0.001	R cerebellum (IV-V-VI)
	-32	-46	-28	97	<0.001	L cerebellum (IV-V-VI)
**Negative connectivity**	-30	-60	-38	534	<0.001	L cerebellum (VII-VIII, CRUS I-II)
	-54	-52	46	330	<0.001	L angular cx(BA 39), L supramarginal cx(BA 40)
	46	-50	36	256	<0.001	R angular cx(BA 39), R supramarginal cx(BA 40)
	-44	14	54	174	<0.001	L frontaleye field (BA 8)
	40	-78	14	165	<0.001	R angular cx(BA 39)
	-32	-82	22	150	<0.001	L angular cx(BA 39)
	32	-64	52	131	<0.001	R SPL (BA 7)

	48	20	46	119	<0.001	R DLPFC (BA 9)
	34	14	60	110	<0.001	R frontaleye field (BA 8)
	8	-72	58	94	<0.001	precuneus
	42	-64	-36	66	<0.001	R cerebellum (CRUS I-II)
	-52	44	2	65	<0.001	L DLPFC (BA 46)
	18	-30	-42	27	<0.05	R cerebellum (X)
**M1_IE_Sup**						
**Positive connectivity**	-20	-34	62	13218	<0.001	B M1 (BA 4), S1(BA 1), SPL (BA 7), precuneus, SMA (BA 6), PM (BA 6), ant and PCC (BA 31-32)
	38	-24	18	851	<0.001	R supramarginal Gyrus (BA 40), R insula (BA 13), R Broca's area (BA 44)
	48	0	4	307	<0.001	R PM (BA 6)
	56	22	32	153	<0.001	R DLPFC (BA 9)
	0	-46	-14	83	<0.001	cerebellum (Vermis IV-V-VI)
	4	-64	-38	68	<0.01	cerebellum (Vermis VIII)
	-18	-42	-26	59	<0.01	L cerebellum (IV-V)
	28	-44	-54	52	<0.01	R cerebellum (VIII)
	-20	-52	-60	51	<0.01	L cerebellum (IX)
	26	-36	-26	36	<0.05	R cerebellum (IV-V)
**Negative connectivity**	48	-44	46	111	<0.001	R supramarginal Gyrus (BA 40)
	10	36	22	94	<0.001	R FEF (BA 8), R ACC (BA 32)
	30	20	-8	83	<0.001	R pars orbitalis (BA 47)
	40	30	16	73	<0.01	R Broca's area (BA 45)
	-8	-84	-16	71	<0.01	L cerebellum (CRUS I)
	-16	2	12	61	<0.01	L caudate
	-40	-52	58	33	<0.05	L supramarginal Gyrus (BA 40)
**M1_IE_Mid**						
**Positive connectivity**	-38	-18	42	15880	<0.001	B M1 (BA 4), S1(BA 1), SPL (BA 7), precuneus, SMA (BA 6), PM (BA 6), ACC and PCC (BA 31-32), Insula (BA 13), primary auditory cx (BA 41), R Putamen
	16	-52	-22	364	<0.001	R cerebellum (IV-V-VI, Vermis IV-V-VI)
	16	-64	-54	149	<0.001	R cerebellum (VIII)
	-12	-60	-20	41	<0.05	L cerebellum (VI)
**Negative connectivity**	46	-42	42	80	<0.001	R supramarginal Gyrus (BA 40)
	16	62	32	49	<0.05	R DLPFC (BA 9)

	20	18	66	54	<0.05	R Superior frontal gyrus
	40	-54	30	62	<0.05	R angular cx (BA 39)
	-20	-88	-32	73	<0.01	L cerebellum CRUS II
**M1_IE_Inf**						
**Positive connectivity**	-58	-2	12	17658	<0.001	B M1 (BA 4), S1(BA 1), SPL (BA 7), precuneus, SMA (BA 6), PM (BA 6), ant and PCC (BA 31-32), Insula (BA 13), primary auditory cx (BA 41), R Putamen,
	-18	-64	-20	202	<0.001	L cerebellum (VI)
	12	-62	-50	196	<0.001	R cerebellum (VIII)
	16	-64	-20	190	<0.001	R cerebellum (VI)
	-12	-62	-52	97	<0.001	L cerebellum (VIII)
**Negative connectivity**						
**Comparison**						
**M1_IE_Sup > M1_Foot**	-22	-34	58	1556	<0.001	L M1 (BA 4), L S1(BA 1), L PM (BA 6)
	22	-26	64	1026	<0.001	R PM (BA 6), R S1, R M1
	-4	-20	50	342	<0.001	SMA (BA 6), ACC (BA 32)
	46	-30	22	121	<0.001	R supramarginal Gyrus (BA 40)
	-40	-22	16	86	<0.001	L S1 (BA 1), L Insula (BA 13)
**M1_Foot > M1_IE_Sup**	-4	-42	74	1271	<0.001	precuneus, L sensory associative cx (BA 5), R S1 (BA 1), R M1 (BA 4)
	2	-94	22	111	<0.001	R Occipital cx (BA 18)
	6	62	30	43	<0.01	R DLPFC (BA 9)
**M1_IE_Sup > M1_Hand**	-20	-34	62	3673	<0.001	B S1 (BA 1), B M1 (BA 4), SMA (BA 6), B SPL (BA 7), precuneous, B ACC and PCC (BA 31-32)
	34	-22	20	179	<0.001	R supramarginal Gyrus (BA 40), R insula (BA 13)
	-16	-44	-52	38	<0.05	L cerebellum (VIII)
	-52	-30	30	37	<0.05	L supramarginal cx (BA 40)
	-22	-34	-26	36	<0.05	R cerebellum (I-IV)
	-30	42	20	33	<0.05	L AntPFC (BA 10)
	34	6	8	27	<0.05	R insula (BA 13)
**M1_Hand > M1_ IE_Sup**	-36	-22	60	1492	<0.001	L PM (BA 6), L S1 (BA 1)
	14	-52	-20	167	<0.001	R cerebellum (V)
	60	-10	38	65	<0.001	R M1 (BA 4)
	-60	-12	38	50	<0.01	L M1 (BA 4)

	64	-50	32	41	<0.05	R angular cx (BA 39)
	0	50	16	35	<0.05	R AntPFC (BA 10)
	56	0	-10	29	<0.05	R sup temporal cx (BA 22)
	4	-56	40	26	<0.05	R PCC (BA 31)
**M1_IE_Sup > M1_Mouth**	-20	-36	62	5135	<0.001	B PM (BA 6), B S1 (BA 1), B ACC (BA 32), SMA (BA 6), precuneous, PCC (BA 31), B SPL (BA 7)
	44	-30	20	167	<0.001	R supramarginal cx (BA 40)
	26	-36	-26	65	<0.001	R cerebellum (I-IV)
	-38	-16	0	35	<0.05	L insula (BA 13)
	36	-18	2	27	<0.05	R insula (BA 13)
	-18	-48	-50	27	<0.05	L cerebellum (IX)
**M1_Mouth > M1_ IE_Sup**	-48	-14	36	2042	<0.001	L S1 (BA 1), L PM (BA 6), L M1(BA 4), L insula (BA 13)
	56	-6	34	1789	<0.001	R S1 (BA 1), R PM (BA 6), R M1(BA 4), R insula (BA 13)
	20	-64	-18	126	<0.001	R cerebellum (V)
	-18	-66	-18	113	<0.001	L cerebellum (V)
**M1_IE_Sup > SMA**	-22	-36	58	706	<0.001	L M1 (BA 4), L S1 (BA 1)
	-40	-4	16	432	<0.001	L PM cx (BA 6), L insula (BA 13)
	22	-32	70	328	<0.001	R M1 (BA 4), R S1 (BA 1)
	44	-62	-38	27	<0.05	R cerebellum CRUS I
	-34	-80	-36	26	<0.05	L cerebellum CRUS II
**SMA > M1_ IE_Sup**	0	-6	54	1422	<0.001	B SMA (BA 6), ACC (BA 32)
	36	0	16	786	<0.001	R insula (BA 13), R Broca area (BA 44), R parsorbitalis (BA 47)
	64	-22	30	270	<0.001	R supramarginal cx (BA 40), R S1 (BA 1)
	-58	-16	18	230	<0.001	L S1 (BA 1), L A1 (BA 41)
	54	4	44	131	<0.001	R PM cx (BA 6)
	42	-32	48	103	<0.001	R S1 (BA 1)
	-38	-16	62	88	<0.001	L M1 (BA 4)
	42	-4	64	63	<0.001	R PM cx (BA 6)
	-26	-50	-18	62	<0.001	L cerebellum (IV-V-VI)
	22	-52	-20	59	<0.001	R cerebellum (IV-V-VI)
	-40	-14	8	46	<0.01	L insula (BA 13)
	-24	-8	74	70	<0.01	L PM cx (BA 6)

	-22	-58	-54	35	<0.05	L cerebellum (VIII)
	-58	-24	44	28	<0.05	L supramarginal cx (BA 40)
	60	8	22	26	<0.05	L Broca area (BA 44)
**M1_IE_Mid > M1_Hand**	-38	-18	42	567	<0.001	L M1 (BA 4),L S1(BA 1)
	38	-16	36	517	<0.001	R M1 (BA 4),R PM (BA 6)
	-52	-2	20	283	<0.001	L PM (BA 6)
	56	0	18	148	<0.001	R PM (BA 6)
	16	-64	18	99	<0.001	precuneus
	16	-30	60	77	<0.001	R M1 (BA 4)
	36	6	8	72	<0.001	R Insula (BA 13)
	-34	4	6	54	<0.01	L Insula (BA 13)
	6	10	36	48	<0.01	R ACC (BA 32)
	18	-50	0	45	<0.01	R Occipital cx (BA 18)
	12	-30	48	44	<0.01	R sensory associative cx (BA 5)
	-4	2	60	43	<0.01	SMA (BA 6)
	8	-44	54	40	<0.01	R PCC (BA 31)
	-8	8	38	40	<0.01	L ACC (BA 32)
	-10	-60	-18	38	<0.01	L cerebellum (V-VI)
	12	-14	42	33	<0.01	R ACC (BA 24)
	-30	42	20	33	<0.01	L AntPFC(BA 10)
	38	-10	12	27	<0.05	R Insula (BA 13)
	-4	-6	66	21	<0.05	SMA (BA 6)
	-10	-14	42	21	<0.05	L ACC (BA 24)
	56	-14	8	20	<0.05	R primary auditory cx (BA 41)
	-38	2	-8	20	<0.05	L Insula (BA 13)
**M1_Hand > M1_IE_Mid**	-36	-24	56	1005	<0.001	L M1 (BA 4),L S1(BA 1),L PM (BA 6)
	2	-60	42	84	<0.001	precuneus
	46	-50	28	53	<0.001	R angular cx(BA 39)
	14	46	50	36	<0.01	R FEF (BA 8)
	-40	-54	34	26	<0.05	L angular cx(BA 39)
	22	-52	-52	26	<0.05	R cerebellum (VIII)
	20	-44	-24	22	<0.05	R cerebellum (VI)

	20	56	36	20	<0.05	R DLPFC (BA 9)
**M1_IE_Mid > M1_Foot**	-38	-18	42	2764	<0.001	L S1 (BA 1), L PM (BA 6), L M1 (BA 4)
	38	-16	38	1912	<0.001	R PM (BA 6), R M1 (BA 4), R S1 (BA 1)
	42	-10	16	150	<0.001	R S1 (BA 1)
	-42	-14	16	145	<0.001	L S1 (BA 1)
	20	-52	-24	114	<0.001	R cerebellum (VI)
	-4	-4	14	50	<0.01	L thalamus (mediodorsal nuclei)
	18	-28	60	40	<0.05	R M1 (BA 4)
	-10	-16	36	28	<0.05	L PCC (BA 23)
**M1_Foot > M1_ IE_Mid**	-4	-42	74	2153	<0.001	precuneous, B SPL (BA 7), B PM (BA 6)
	-16	-38	-54	55	<0.01	L cerebellum (VIII)
	28	-38	-52	35	<0.05	R cerebellum (VIII)
	4	-94	20	28	<0.05	R secondary visual area (BA 18)
**M1_ IE_Mid > M1_Mouth**	-36	-18	42	907	<0.001	L M1 (BA 4), L PM (BA 6), L S1 (BA 1)
	-12	-14	42	759	<0.001	B ACC (BA 32, BA 24), SMA (BA 6), B PM (BA 6), B PCC (BA 31),
	46	-8	50	145	<0.001	R PM (BA 6)
	20	-28	58	136	<0.001	R M1 (BA 4)
	38	14	6	97	<0.001	R Broca area (BA 44), R insula (BA 13)
	14	-42	52	79	<0.001	precuneous
	-34	4	8	45	<0.01	L insula (BA 13)
	12	-6	72	39	<0.01	R PM (BA 6)
	-6	-10	68	37	<0.05	SMA (BA 6)
	6	-64	-18	35	<0.05	cerebellum vermis (VI)
**M1_Mouth > M1_ IE_Mid**	-48	-14	36	982	<0.001	L M1 (BA 4), L PM (BA 6), L S1 (BA 1)
	54	-6	32	777	<0.001	R S1 (BA 1), R M1 (BA 4)
	-40	8	54	26	<0.05	L PM (BA 6)
**M1_ IE_Mid > SMA**	-38	-18	42	1229	<0.001	B M1 (BA 4)
	36	-18	38	1048	<0.001	B PM (BA 6), SMA (BA 6)
	30	-76	-40	232	<0.001	R SPL (BA 7)
	28	-50	-58	59	<0.001	R cerebellum (VIII)
	-48	32	-2	56	<0.001	L Broca area (BA 45)
	18	-86	-20	55	<0.001	L cerebellum (CRUS I)

	-36	-82	-36	48	<0.01	L cerebellum (CRUS II)
	-40	16	30	35	<0.05	L Broca area (BA 44)
	6	-60	16	29	<0.05	R PCC (BA 31)
**SMA > M1_ IE_Mid**	0	-8	54	1876	<0.001	B ACC (BA 32), B PM (BA 6), SMA (BA 6), L SPL (BA 7), precuneous, PCC (BA 31)
	64	-24	30	443	<0.001	R supramarginal cx (BA 40)
	42	2	14	213	<0.001	R PM (BA 6)
	-50	-30	22	198	<0.001	L supramarginal cx (BA 40)
	-48	0	6	160	<0.001	L PM (BA 6)
	26	-46	70	115	<0.001	R SPL (BA 7)
	50	20	-4	87	<0.001	R pars orbitalis (BA 47)
	-40	-2	-2	86	<0.001	L insula (BA 13)
	-30	-50	-56	62	<0.001	L cerebellum (VIII)
	42	-38	68	28	<0.05	R S1 (BA 1)
	-36	20	10	27	<0.05	L Broca area (BA 45)
	38	-14	-2	26	<0.05	R insula (BA 13)
**M1_IE_Inf > M1_Foot**	62	2	14	3925	<0.001	RM1 (BA 4), R PM (BA 6), R S1 (BA 1), R insula (BA 13), R A1 (BA 41), R supramarginal cx (BA 40), R sup temporal cx (BA 22)
	-58	-2	16	3630	<0.001	L M1 (BA 4), L PM (BA 6), L S1 (BA 1), L insula (BA 13), L A1 (BA 41), L supramarginal cx (BA 40), L sup temporal cx (BA 22)
	-4	-4	56	90	<0.001	SMA (BA 6)
	-14	-60	-16	87	<0.001	L cerebellum (IV-V-VI)
	12	-64	-48	61	<0.01	R cerebellum (VIII-IX)
	4	-6	16	37	<0.05	R thalamus (Mediodorsal nucleus)
	44	4	-16	29	<0.05	R temporal cx (BA 22)
	16	-64	-22	28	<0.05	R cerebellum (VI)
**M1_Foot > M1_IE_Inf**	-4	-42	72	2233	<0.001	R SPL (BA 7), B S1 (BA 1), B M1 (BA 4), precuneous
	-36	-74	26	61	<0.01	L secondary visual cx (BA 19)
	-14	-42	-58	48	<0.01	L cerebellum (VIII-IX)
	16	-46	-56	37	<0.05	R cerebellum (VIII-IX)
	-48	-62	-44	60	<0.05	L cerebellum (CRUS II)
**M1_IE_Inf > M1_Hand**	-54	-4	14	2205	<0.001	L PM (BA 6), L S1 (BA 1), L insula (BA 13), L Broca area (BA 44), L A1 (BA 41), L temporal cx (BA 22)
	62	2	14	2187	<0.001	R PM (BA 6), R S1 (BA 1), R insula (BA 13), R Broca area (BA 44), R A1 (BA 41), R temporal cx (BA 22), R supramarginal cx (BA 40)

	-16	-60	-16	175	<0.001	L cerebellum (VI)
	-4	14	34	148	<0.001	B ACC (BA 32)
	64	-26	36	143	<0.001	R supramarginal cx (BA 40)
	4	4	54	119	<0.001	SMA (BA 6)
	-56	-36	50	118	<0.001	L supramarginal cx (BA 40)
	-34	36	16	100	<0.001	L AntPFC (BA 10)
	16	-64	18	36	<0.05	Precuneous cx
	-42	34	22	30	<0.05	L DLPFC (BA 46)
**M1_Hand > M1_IE_Inf**	-34	-26	56	1626	<0.001	L M1 (BA 4), L PM (BA 6)
	-6	-46	42	144	<0.001	Precuneous cx, PCC (BA 31)
	22	-46	-26	79	<0.001	R cerebellum (IV-V-VI)
	36	-22	54	55	<0.01	R M1 (BA 4)
	16	-86	-38	49	<0.01	R cerebellum (CRUS II)
	-44	-54	32	25	<0.05	L angular cx(BA 39)
**M1_IE_Inf > M1_Mouth**	-56	-2	12	1611	<0.001	L insula (BA 13), L PM (BA 6)
	64	6	4	1167	<0.001	R PM (BA 6), R Insula (BA 13), R Broca's area (BA 44), R primary auditory cx (BA 41), R sup temporal cx(BA 22)
	-10	4	38	620	<0.001	ACC (BA 32), SMA (BA 6)
	62	-30	34	335	<0.001	R supramarginal cx(BA 40)
	-46	-38	20	131	<0.001	L sup temporal cx(BA 22)
	36	46	36	52	<0.01	R DLPFC (BA 9)
	-20	-2	18	43	<0.01	L caudate
	44	-2	46	30	<0.05	R PM (BA 6)
**M1_Mouth > M1_IE_Inf**	-48	-14	36	709	<0.001	L S1 (BA 1), L M1 (BA 4)
	48	-10	34	409	<0.001	R M1 (BA 4)
	-12	44	40	112	<0.001	L FEF (BA 8)
	44	-70	-40	75	<0.001	R cerebellum (CRUS I)
	-44	-52	36	43	<0.01	L angular cx(BA 39)
	-4	-48	36	32	<0.05	L PCC (BA 23)
	-40	-34	60	28	<0.05	L S1 (BA 1)
	-8	32	56	24	<0.05	L FEF (BA 8)
**M1_IE_Inf > SMA**	-56	-6	14	1571	<0.001	L PM (BA 6), L M1 (BA 4), L S1(BA 1), L Broca area (BA 44), L sup temporal cx (BA 22)

	60	-2	18	1506	<0.001	R PM (BA 6),R M1 (BA 4), R S1(BA 1), R Broca area (BA 44), R sup temporal cx (BA 22)
	36	-10	14	59	<0.01	R insula cx (BA 13)
	-36	-10	12	57	<0.01	L insula cx (BA 13)
	-48	34	2	44	<0.01	L pars triangularis (BA 45)
	-14	-86	-8	40	<0.01	L secondary visual area (BA 18)
	-14	-60	-16	40	<0.01	L cerebellum (VI)
	-26	-66	-14	37	<0.05	L secondary visual area (BA 19)
	-44	14	20	34	<0.05	L Broca area (BA 44)
	-60	-42	10	34	<0.05	L supramarginal cx (BA 40)
	-64	-26	6	33	<0.05	L mid temporal cx (BA 21)
	-44	-36	14	32	<0.05	L sup temporal cx (BA 22)
	64	-24	-18	25	<0.05	R mid temporal cx (BA 21)
	12	-64	-42	22	<0.05	R cerebellum (VIII)
**SMA > M1_IE_Inf**	0	-6	54	2726	<0.001	B PM (BA 6), SMA (BA 6), B SPL (BA 7), B S1 (BA 1), B ACC (BA 32), precuneous
	16	-16	72	143	<0.001	R PM (BA 6)
	22	-52	-54	80	<0.001	R cerebellum (VIII)
	-42	-24	20	44	<0.01	L supramarginal cx (BA 40)
	14	-44	56	29	<0.05	R SPL (BA 7)
	34	-38	64	27	<0.05	R S1 (BA 1)
	6	18	34	26	<0.05	B ACC (BA 32)
	36	22	-10	26	<0.05	R pars orbitalis (BA 47)
	30	-36	-30	24	<0.05	R cerebellum (IV-V-VI)
**M1_IE_Mid > M1_IE_Sup**	-38	-18	42	1781	<0.001	L PM (BA 6), L S1 (BA 1)
	48	-8	44	1423	<0.001	R PM (BA 6), R M1 (BA 4), R S1 (BA 1),
	16	-54	-22	81	<0.001	R cerebellum (IV-V-VI)
	-34	-8	12	34	<0.05	L M1 (BA 4)
	-38	0	42	28	<0.01	L PM (BA 6)
**M1_IE_Sup > M1_IE_Mid**	-22	-36	58	2984	<0.001	B S1 (BA 1), B M1 (BA 4), B PM (BA 6), SMA (BA 6), B PCC (BA 31), B SPL (BA 7), L Secondary sensory cx (BA 5), precuneous
	46	-26	26	40	<0.01	R supramarginal cx (BA 40)
	32	-34	-34	38	<0.05	R cerebellum (IV-V-VI)
	8	-4	44	35	<0.05	B ACC (BA 32), SMA (BA 6)

	-18	-46	-52	32	<0.05	L cerebellum (IX)
	34	-22	22	25	<0.05	R S1 (BA 1)
	-8	-16	78	24	<0.05	L PM (BA 6), SMA (BA 6)
**M1_IE_Inf > M1_IE_Sup**	-54	-8	14	2955	<0.001	L M1 (BA 4), L S1 (BA 1), L PM cx (BA 6), L supramarginal cx (BA 40), L sup temporal cx (BA 22), L A1 (BA 41)
	62	2	14	2885	<0.001	R M1 (BA 4), R S1 (BA 1), R PM cx (BA 6), R supramarginal cx (BA 40), R sup temporal cx (BA 22), R A1 (BA 41)
	-18	-66	-18	192	<0.001	L cerebellum (VI)
	22	-66	-20	109	<0.001	R cerebellum (VI)
	-60	-22	46	68	<0.001	L supramarginal cx(BA 40)
	-6	4	52	40	<0.01	SMA (BA 6)
	12	-62	-50	36	<0.05	R cerebellum (IV-V-VI)
	46	-42	18	32	<0.05	R supramarginal cx (BA 40)
	-18	4	14	32	<0.05	L caudate
	46	-26	-2	26	<0.05	R superior temporal cx (BA 22)
**M1_IE_Sup > M1_IE_Inf**	-20	-34	62	3884	<0.001	B S1 (BA 1), B M1 (BA 4), B PM (BA 6), SMA (BA 6), B ACC (BA 32), L associative sensory cx (BA 5)
	26	-36	-28	39	<0.01	R cerebellum (VIII)
**M1_IE_Inf > M1_IE_Mid**	-54	-8	12	1663	<0.001	L PM (BA 6), L S1 (BA 1), L insula (BA 13), L sup temporal cx (BA 22), L A1 (BA 41)
	40	4	-6	720	<0.001	R PM (BA 6), R S1 (BA 1), R insula (BA 13), R sup temporal cx (BA 22),R A1 (BA 41)
	60	-14	26	294	<0.001	R supramarginal cx(BA 40)
	-44	-38	18	36	<0.01	L supramarginal cx(BA 40)
**M1_IE_Mid > M1_IE_Inf**	-38	-18	44	889	<0.001	L M1 (BA4), L S1 (BA 1)
	38	-16	42	144	<0.001	R M 1 (BA 4)
	6	58	10	26	<0.05	R AntPFC (BA 10)
	16	-30	62	24	<0.05	R M1 (BA 4)
**M1_Hand > M1_Foot**	-36	-24	60	2163	<0.001	L S1 (BA 1), L M1 (BA 4), L PM cx (BA 6)
	56	-12	46	543	<0.001	R PM cx (BA 6)
	20	-48	-26	226	<0.001	R cerebellum (IV-V-VI)
	-56	-2	34	83	<0.001	L PM cx (BA 6)
	-46	-18	20	64	<0.001	L S1 (BA 1)
	12	-58	-42	57	<0.01	R PM cx (BA 6)
	-8	-10	54	49	<0.01	SMA (BA 6)

**M1_Foot > M1_Hand**	-4	-42	72	2685	<0.001	precuneous, B sensory associative cx (BA 5), B M1 (BA 4), B SPL (BA 7), R S1 (BA 1)
	-14	-40	-56	85	<0.001	L cerebellum (VIII-IX)
	-36	46	24	47	<0.001	L AntPFC (BA 10)
	18	-70	34	38	<0.05	precuneous
	26	-28	-30	37	<0.05	R cerebellum (IV-V)
	4	-90	32	35	<0.05	R secondary visual cx (BA 19)
	28	40	20	26	<0.05	R AntPFC (BA 10)
**M1_Hand > M1_Mouth**	-36	-24	58	1351	<0.001	L PM cx (BA 6), L M1 (BA 4)
	-8	-8	50	190	<0.001	SMA (BA 6)
	20	-44	-24	89	<0.001	R cerebellum (IV-V)
	22	-54	-54	32	<0.01	R cerebellum (VIII)
	56	-52	26	24	<0.05	R angular cx (BA 39)
	28	-20	50	21	<0.05	R M1 (BA 4)
**M1_Mouth > M1_Hand**	-46	-14	36	1141	<0.001	L PM cx (BA 6), L M1 (BA 4)
	54	-6	34	1082	<0.001	R PM cx (BA 6), R S1 (BA 1), R M1 (BA 4)
	38	-4	8	81	<0.001	R insula (BA 13)
	-14	-66	-18	76	<0.001	L cerebellum (VI)
	-46	-52	58	58	<0.001	L supramarginal cx(BA 40)
	-36	-6	10	46	<0.01	L insula (BA 13)
	52	-36	58	43	<0.01	R supramarginal cx(BA 40)
	-42	10	54	21	<0.05	L PM cx (BA 6)
	-42	34	24	20	<0.05	R DLPFC (BA 9)
**M1_Foot > M1_Mouth**	-4	-42	72	2558	<0.001	precuneous, B SPL (BA 7), B SMA (BA 6), PCC (BA 31),
	16	-44	-52	63	<0.01	R cerebellum (VI)
	-14	-42	-60	62	<0.01	L cerebellum (VIII-IX)
	4	4	46	50	<0.01	B ACC (BA 32), SMA (BA 6)
	24	-34	-26	48	<0.01	R cerebellum (IV-V)
	0	-96	8	47	<0.01	R secondary visual cx (BA 18)
	36	46	36	43	<0.01	R DLPFC (BA 9)
**M1_Mouth > M1_Foot**	-48	-14	36	2565	<0.001	L S1 (BA 1), L M1 (BA 4), L insula (BA 13), L A1 (BA 41)
	62	-6	32	2453	<0.001	R M1 (BA 4), R S1 (BA 1), R PM cx (BA 6), R supramarginal cx (BA 40)
	20	-54	-20	82	<0.001	R cerebellum (IV-V)

	-14	-58	-18	57	<0.01	L cerebellum (VI)
	28	-12	-16	26	<0.05	R hippocampus
**M1_Foot > SMA**	-4	-40	74	1215	<0.001	precuneous, B S1 (BA 1), B SPL (BA 7)
	20	-72	-34	132	<0.001	R cerebellum (CRUS I-II)
	6	-88	30	99	<0.001	R secondary visual area (BA 18)
	-36	-74	26	93	<0.001	L angular cx (BA 39)
	-46	-64	-44	77	<0.001	L cerebellum (CRUS II)
	-32	30	52	68	<0.01	L FEF (BA 8)
	48	-70	-16	60	<0.01	R cerebellum (CRUS I)
	36	-64	-48	53	<0.01	R cerebellum (VIII- IX)
	-4	-48	44	48	<0.01	precuneous
	-22	-78	-38	45	<0.01	L cerebellum (CRUS I-II)
	-56	-56	-6	38	<0.05	L fusiform cx (BA 37)
	-52	-72	-14	35	<0.05	L secondary visual area (BA 19)
	6	62	32	31	<0.05	R DLPFC (BA 9)
	-52	42	2	31	<0.05	R DLPFC (BA 46)
	-10	-40	-50	31	<0.05	L cerebellum (IX)
**SMA > M1_Foot**	-2	-6	54	4047	<0.001	B PM (BA 6), B FEF (BA 8), B ACC (BA 32), SMA (BA 6), B S1 (BA 1), B PCC (BA 31)
	40	2	12	2156	<0.001	R insula (BA 13), R, M1 (BA 4), R PM (BA 6), R putamen, R pallidum, R broca area (BA 44), R pars orbitalis (BA 47)
	-38	-4	14	1555	<0.001	L insula (BA 13), L M1 (BA 4), L PM (BA 6), L putamen, L pallidum, L broca area (BA 44), L pars orbitalis (BA 47)
	42	-38	66	576	<0.001	R S1 (BA 1), R supramarginal cx (BA 40)
	22	-52	-22	99	<0.001	R cerebellum (IV-V-VI)
	2	-6	16	62	<0.01	R thalamus (Mediodorsal nucleus)
	-58	2	34	57	<0.01	L cerebellum (VII-VIII, CRUS II)
	24	-54	-52	51	<0.01	R cerebellum (VIII-IX)
	56	-16	44	49	<0.01	R S1 (BA 1)
	12	-50	-18	40	<0.05	R cerebellum (IV-V)
	-56	-24	44	37	<0.05	R S1 (BA 1)
**M1_Hand > SMA**	-36	-24	56	1112	<0.001	L M1 (BA 4)
	38	-84	-32	193	<0.001	R cerebellum (CRUS I-II)
	-50	-54	34	154	<0.001	L angular cx (BA 39)

	-20	22	46	107	<0.001	R S1 (BA 1), R SPL (BA 7)
	-24	-88	-34	97	<0.001	L cerebellum (CRUS I-II)
	16	46	48	87	<0.001	R FEF (BA 8)
	-56	-10	32	84	<0.001	L M1 (BA 4), L S1 (BA 1)
	54	-52	26	83	<0.001	R angular cx (BA 39)
	40	-68	-40	62	<0.001	R cerebellum (CRUS I-II)
	62	-14	-12	49	<0.01	R sup temporal cx (BA 22)
	66	-2	22	43	<0.01	R M1 (BA 4), R PM (BA 6)
	-12	-82	-40	95	<0.01	L cerebellum (CRUS II)
	-38	16	30	22	<0.05	L DLPFC (BA 9)
**SMA > M1_Hand**	0	-4	54	3831	<0.001	B ACC (BA 32), SMA (BA 6), B PM (BA 6), R FEF (BA 8), precuneous, B PCC (BA 31), L SPL (BA 7)
	38	6	10	1483	<0.001	R insula (BA 13), R PM (BA 6), R putamen, R pallidum, R broca area (BA 44), R pars orbitalis (BA 47)
	-36	4	6	976	<0.001	L insula (BA 13), L PM (BA 6), L putamen, L broca area (BA 44), L pars orbitalis (BA 47)
	56	-28	32	856	<0.001	R supramarginal cx (BA 40), R S1 (BA 1), R insula (BA 13)
	-56	-34	36	284	<0.001	L supramarginal cx (BA 40), L S1 (BA 1)
	28	-46	72	137	<0.001	R SPL (BA 7), R S1 (BA 1)
	-30	38	20	102	<0.001	L AntPFC (BA 10)
	32	34	30	94	<0.001	R DLPFC (BA 9)
	-28	-48	-28	79	<0.001	L cerebellum (IV-V-VI)
	32	-42	44	33	<0.05	R cerebellum (CRUS I)
	-30	-50	-56	32	<0.05	L cerebellum (VIII)
	54	-32	58	30	<0.05	R supramarginal cx (BA 40)
	36	-48	-26	26	<0.05	R cerebellum (VI)
	-62	-16	14	23	<0.05	L supramarginal cx (BA 40)
**M1_Mouth > SMA**	-48	-14	36	1258	<0.001	L M1 (BA 4), L S1 (BA 1), L PM (BA 6)
	54	-6	34	1063	<0.001	R M1 (BA 4), R S1 (BA 1), R PM (BA 6)
	60	-28	32	663	<0.001	R supramarginal cx (BA 40)
	32	-66	-36	312	<0.001	R cerebellum (VII-VIII, CRUS I-II)
	-44	6	50	244	<0.001	R FEF (BA 8)
	-50	-54	34	204	<0.001	L angular cx (BA 39)
	-42	-70	-32	144	<0.001	L cerebellum (CRUS I-II)

	-48	34	-2	113	<0.001	L pars orbitalis (BA 47), L pars triangularis (BA 45)
	-10	18	62	93	<0.001	L FEF (BA 8)
	-22	50	36	36	<0.01	L DLPFC (BA 9)
	-30	-86	-30	36	<0.01	L cerebellum (CRUS I-II)
	38	-60	40	29	<0.05	R angular cx (BA 39)
	20	-72	54	28	<0.05	R SPL (BA 7)
	18	-58	-22	27	<0.05	R cerebellum (VI)
	64	-24	-18	26	<0.05	R middle temporal cx (BA 21)
	16	-84	-22	23	<0.05	R cerebellum (CRUS I)
	-12	-74	60	20	<0.05	L SPL (BA 7)
	16	-82	-38	19	<0.05	R cerebellum (CRUS II)
**SMA > M1_Mouth**	0	-8	54	4723	<0.001	B ACC (BA 32), SMA (BA 6), B PM (BA 6), R FEF (BA 8), precuneous, B PCC (BA 31), L SPL (BA 7)
	36	14	8	929	<0.001	R insula (BA 13), R PM (BA 6), R putamen, broca area (BA 44)
	-38	-16	4	790	<0.001	L insula (BA 13), L PM (BA 6), L putamen, L broca area (BA 44)
	-48	-30	22	237	<0.001	L supramarginal cx (BA 40), L S1 (BA 1)
	38	-14	-2	174	<0.001	R insula (BA 13), R putamen
	32	-42	-50	113	<0.001	R cerebellum (VIII-IX)
	-28	-50	-56	97	<0.001	L cerebellum (VIII-IX)
	32	44	32	92	<0.001	R DLPFC (BA 9)
	24	-38	68	51	<0.01	R S1 (BA 1), R SPL (BA 7)
	22	-38	-26	27	<0.05	R cerebellum (IV-V)
	-38	-8	54	25	<0.05	L PM (BA 6)
	-28	36	20	20	<0.05	L AntPFC (BA 10)
	28	-44	-28	20	<0.05	R cerebellum (VI)

Note: ***cluster forming threshold p<.001, cluster-level p-FDR<.05.*** *R: right, L: left, B: bilateral, BA: Brodmann area, cx: cortex, AntPFC: anterior prefrontal cortex, S1: primary sensory cortex, M1: primary motor cortex, PM : premotor cortex, SMA: supplementary motor area, FEF: frontal eye field, ACC: anterior cingulate cortex, PCC: posterior cingulate cortex, DLPFC: dorsolateral prefrontal cortex, SPL: superior parietal cortex, A1: primary auditory cortex, M1_Inf_IE: inferior intereffector, M1_Mid_IE: middle intereffector, M1_Sup_IE: superior intereffector.*

SI Appendix B: Replication of the seed-to-voxels rs-FC in the healthy controls

SI Appendix B1: Effectors and intereffectors seed-to-voxels rs-FC in healthy controls

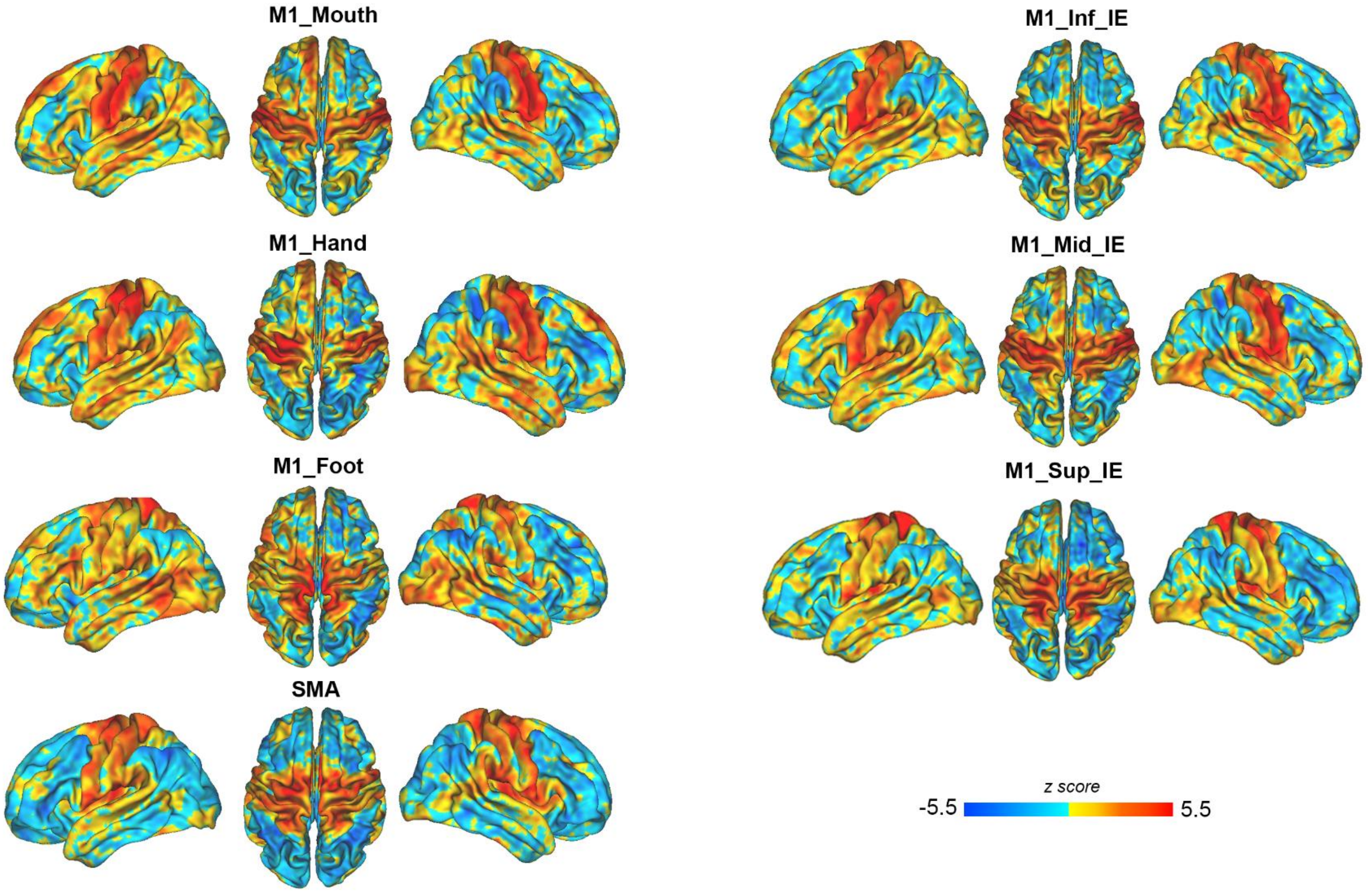

Note: This figure displays unthresholded Rs-FC maps of the 7 seeds: 3 intereffectors (M1_Mouth, M1-Hand, M1_Foot), 3 effectors (M1_inf_IE, M1_Mid_IE, M1_Sup_IE) and SMA in HC. The color scale reflects positive (yellow-red) and negative (blue) connectivity between the seed and the whole brain. Darker areas reflect stronger rs-FC *M1_Inf_IE: inferior intereffector, M1_Mid_IE: middle intereffector, M1_Sup_IE: superior intereffector, SMA: supplementary motor area, M1: primary motor area.*

SI Appendix B2: Cluster statistics related to the seed-to-voxels analysis with M1 effectors and intereffectors within the healthy controls.

	x	y	z	size	p-FDR	Brain Areas
**M1_Foot**						
**Positive connectivity**	-4	-42	72	5773	<0.001	B M1 (BA 4), S1(BA 1), SPL (BA 7), precuneus, SMA (BA 6), PM (BA 6), ant and PCC (BA 31-32)
	18	-44	-58	129	<0.001	R cerebellum (VIII)
	38	-20	20	69	<0.01	R Insula (BA 13)
	-14	-42	-58	65	<0.01	L cerebellum (VIII-IX)
	22	-30	-26	57	<0.01	R cerebellum (IV-V)
	44	-72	-46	50	<0.01	R cerebellum (CRUS II)
	2	-94	20	44	<0.05	R Occipital cx (BA 18)
	-52	-30	18	34	<0.05	L supramarginal Gyrus (BA 40)
	-32	-20	18	32	<0.05	L Insula (BA 13)
**Negative connectivity**	54	-40	54	342	<0.001	R supramarginal Gyrus (BA 40)
	-8	8	16	94	<0.001	L caudate
	44	18	42	53	<0.01	R FEF (BA 8)
	44	-66	48	31	<0.05	R angular cx(BA 39)
**M1_Hand**						
**Positive connectivity**	-36	-24	56	9669	<0.001	B M1 (BA 4), S1(BA 1), SPL (BA 7), precuneus, SMA (BA 6), PM (BA 6), ACC and PCC (BA 31-32)
	16	-52	-22	348	<0.001	R cerebellum (IV-V-VI)
	40	-12	16	316	<0.001	R S1 (BA1), R supramarginal Gyrus (BA 40), R Insula (BA 13)
	22	-54	-54	274	<0.001	R cerebellum (VIII-IX, vermis VIII)
	30	-88	-36	49	<0.01	R cerebellum (CRUS II)
	-4	50	-2	36	<0.05	B AntPFC(BA 10)
**Negative connectivity**	-60	-40	48	370	<0.001	L supramarginal Gyrus (BA 40)
	52	-40	54	273	<0.001	R supramarginal Gyrus (BA 40), R SPL (BA 7)
	6	-60	68	174	<0.001	precuneous

	18	-10	16	60	<0.01	R thalamus (ventral anterior nucleus)
	-10	6	8	49	<0.01	L caudate
	36	16	62	40	<0.05	R FEF (BA 8)
	-30	-70	-24	40	<0.05	L cerebellum (VI)
**M1_Mouth**						
**Positive connectivity**	-48	-14	36	10884	<0.001	B M1 (BA 4),S1(BA 1),SPL (BA 7), precuneus, SMA (BA 6), PM (BA 6), ACC and PCC (BA 31-32)
	18	-58	-24	205	<0.001	R cerebellum (VI)
	12	-64	-46	153	<0.001	R cerebellum (VIII)
	-16	-66	-18	148	<0.001	L cerebellum (VI)
	-14	48	34	121	<0.001	L DLPFC 8 (BA 9)
	-4	-4	56	86	<0.001	SMA (BA 6)
	-12	-64	-50	68	<0.01	L cerebellum (VIII)
	-4	12	64	57	<0.01	L PM (BA 6)
**Negative connectivity**	34	50	34	90	<0.001	R DLPFC (BA 9)
	8	36	30	59	<0.01	R FEF (BA 8), R ACC (BA 32)
	16	-8	16	43	<0.05	R thalamus (ventral anterior nucleus)
	-14	0	14	39	<0.05	L caudate
	12	50	24	30	<0.05	R AntPFC(BA 10)
**SMA**						
**Positive connectivity**	0	-8	54	20641	<0.001	B M1 (BA 4),S1(BA 1),SPL (BA 7), precuneus, SMA (BA 6), PM (BA 6), ACC and PCC (BA 31-32)
	24	-54	-54	376	<0.001	R cerebellum (VIII-IX)
	-28	-50	-54	238	<0.001	L cerebellum (VIII-IX)
	28	-46	-26	203	<0.001	R cerebellum (IV-V-VI)
	-32	-46	-28	97	<0.001	L cerebellum (IV-V-VI)
**Negative connectivity**	-30	-60	-38	534	<0.001	L cerebellum (VII-VIII, CRUS I-II)
	-54	-52	46	330	<0.001	L angular cx(BA 39), L supramarginal cx(BA 40)
	46	-50	36	256	<0.001	R angular cx(BA 39), R supramarginal cx(BA 40)
	-44	14	54	174	<0.001	L frontaleye field (BA 8)
	40	-78	14	165	<0.001	R angular cx(BA 39)
	-32	-82	22	150	<0.001	L angular cx(BA 39)
	32	-64	52	131	<0.001	R SPL (BA 7)

	48	20	46	119	<0.001	R DLPFC (BA 9)
	34	14	60	110	<0.001	R frontaleye field (BA 8)
	8	-72	58	94	<0.001	precuneus
	42	-64	-36	66	<0.001	R cerebellum (CRUS I-II)
	-52	44	2	65	<0.001	L DLPFC (BA 46)
	18	-30	-42	27	<0.05	R cerebellum (X)
**M1_IE_Sup**						
**Positive connectivity**	-20	-34	62	13218	<0.001	B M1 (BA 4), S1(BA 1), SPL (BA 7), precuneus, SMA (BA 6), PM (BA 6), ant and PCC (BA 31-32)
	38	-24	18	851	<0.001	R supramarginal Gyrus (BA 40), R insula (BA 13), R Broca's area (BA 44)
	48	0	4	307	<0.001	R PM (BA 6)
	56	22	32	153	<0.001	R DLPFC (BA 9)
	0	-46	-14	83	<0.001	cerebellum (Vermis IV-V-VI)
	4	-64	-38	68	<0.01	cerebellum (Vermis VIII)
	-18	-42	-26	59	<0.01	L cerebellum (IV-V)
	28	-44	-54	52	<0.01	R cerebellum (VIII)
	-20	-52	-60	51	<0.01	L cerebellum (IX)
	26	-36	-26	36	<0.05	R cerebellum (IV-V)
**Negative connectivity**	48	-44	46	111	<0.001	R supramarginal Gyrus (BA 40)
	10	36	22	94	<0.001	R FEF (BA 8), R ACC (BA 32)
	30	20	-8	83	<0.001	R pars orbitalis (BA 47)
	40	30	16	73	<0.01	R Broca's area (BA 45)
	-8	-84	-16	71	<0.01	L cerebellum (CRUS I)
	-16	2	12	61	<0.01	L caudate
	-40	-52	58	33	<0.05	L supramarginal Gyrus (BA 40)
**M1_IE_Mid**						
**Positive connectivity**	-38	-18	42	15880	<0.001	B M1 (BA 4), S1(BA 1), SPL (BA 7), precuneus, SMA (BA 6), PM (BA 6), ACC and PCC (BA 31-32), Insula (BA 13), primary auditory cx (BA 41), R Putamen
	16	-52	-22	364	<0.001	R cerebellum (IV-V-VI, Vermis IV-V-VI)
	16	-64	-54	149	<0.001	R cerebellum (VIII)
	-12	-60	-20	41	<0.05	L cerebellum (VI)
**Negative connectivity**	46	-42	42	80	<0.001	R supramarginal Gyrus (BA 40)

**M1_IE_Inf**						
**Positive connectivity**	-58	-2	12	17658	<0.001	B M1 (BA 4), S1(BA 1), SPL (BA 7), precuneus, SMA (BA 6), PM (BA 6), ant and PCC (BA 31-32), Insula (BA 13), primary auditory cx (BA 41), R Putamen,
	-18	-64	-20	202	<0.001	L cerebellum (VI)
	12	-62	-50	196	<0.001	R cerebellum (VIII)
	16	-64	-20	190	<0.001	R cerebellum (VI)
	-12	-62	-52	97	<0.001	L cerebellum (VIII)
**Negative connectivity**	16	62	32	49	<0.05	R DLPFC (BA 9)
**Comparison**						
**M1_IE_Sup > M1_Foot**	-22	-34	58	1556	<0.001	L M1 (BA 4), L S1(BA 1), L PM (BA 6)
	22	-26	64	1026	<0.001	R PM (BA 6), R S1, R M1
	-4	-20	50	342	<0.001	SMA (BA 6), ACC (BA 32)
	46	-30	22	121	<0.001	R supramarginal Gyrus (BA 40)
	-40	-22	16	86	<0.001	L S1 (BA 1), L Insula (BA 13)
**M1_Foot > M1_IE_Sup**	-4	-42	74	1271	<0.001	precuneus, L sensory associative cx (BA 5), R S1 (BA 1), R M1 (BA 4)
	2	-94	22	111	<0.001	R Occipital cx (BA 18)
	6	62	30	43	<0.01	R DLPFC (BA 9)
**M1_IE_Sup > M1_Hand**	-20	-34	62	3673	<0.001	B S1 (BA 1), B M1 (BA 4), SMA (BA 6), B SPL (BA 7), precuneous, B ACC and PCC (BA 31-32)
	34	-22	20	179	<0.001	R supramarginal Gyrus (BA 40), R insula (BA 13)
	-16	-44	-52	38	<0.05	L cerebellum (VIII)
	-52	-30	30	37	<0.05	L supramarginal cx (BA 40)
	-22	-34	-26	36	<0.05	R cerebellum (I-IV)
	-30	42	20	33	<0.05	L AntPFC (BA 10)
	34	6	8	27	<0.05	R insula (BA 13)
**M1_Hand > M1_ IE_Sup**	-36	-22	60	1492	<0.001	L PM (BA 6), L S1 (BA 1)
	14	-52	-20	167	<0.001	R cerebellum (V)
	60	-10	38	65	<0.001	R M1 (BA 4)
	-60	-12	38	50	<0.01	L M1 (BA 4)
	64	-50	32	41	<0.05	R angular cx (BA 39)
	0	50	16	35	<0.05	R AntPFC (BA 10)

	56	0	-10	29	<0.05	R sup temporal cx (BA 22)
	4	-56	40	26	<0.05	R PCC (BA 31)
**M1_IE_Sup > M1_Mouth**	-20	-36	62	5135	<0.001	B PM (BA 6), B S1 (BA 1), B ACC (BA 32), SMA (BA 6), precuneous, PCC (BA 31), B SPL (BA 7)
	44	-30	20	167	<0.001	R supramarginal cx (BA 40)
	26	-36	-26	65	<0.001	R cerebellum (I-IV)
	-38	-16	0	35	<0.05	L insula (BA 13)
	36	-18	2	27	<0.05	R insula (BA 13)
	-18	-48	-50	27	<0.05	L cerebellum (IX)
**M1_Mouth > M1_ IE_Sup**	-48	-14	36	2042	<0.001	L S1 (BA 1), L PM (BA 6), L M1(BA 4), L insula (BA 13)
	56	-6	34	1789	<0.001	R S1 (BA 1), R PM (BA 6), R M1(BA 4), R insula (BA 13)
	20	-64	-18	126	<0.001	R cerebellum (V)
	-18	-66	-18	113	<0.001	L cerebellum (V)
**M1_IE_Sup > SMA**	-22	-36	58	706	<0.001	L M1 (BA 4), L S1 (BA 1)
	-40	-4	16	432	<0.001	L PM cx (BA 6), L insula (BA 13)
	22	-32	70	328	<0.001	R M1 (BA 4), R S1 (BA 1)
	44	-62	-38	27	<0.05	R cerebellum CRUS I
	-34	-80	-36	26	<0.05	L cerebellum CRUS II
**SMA > M1_ IE_Sup**	0	-6	54	1422	<0.001	B SMA (BA 6), ACC (BA 32)
	36	0	16	786	<0.001	R insula (BA 13), R Broca area (BA 44), R parsorbitalis (BA 47)
	64	-22	30	270	<0.001	R supramarginal cx (BA 40), R S1 (BA 1)
	-58	-16	18	230	<0.001	L S1 (BA 1), L A1 (BA 41)
	54	4	44	131	<0.001	R PM cx (BA 6)
	42	-32	48	103	<0.001	R S1 (BA 1)
	-38	-16	62	88	<0.001	L M1 (BA 4)
	42	-4	64	63	<0.001	R PM cx (BA 6)
	-26	-50	-18	62	<0.001	L cerebellum (IV-V-VI)
	22	-52	-20	59	<0.001	R cerebellum (IV-V-VI)
	-40	-14	8	46	<0.01	L insula (BA 13)
	-24	-8	74	70	<0.01	L PM cx (BA 6)
	-22	-58	-54	35	<0.05	L cerebellum (VIII)
	-58	-24	44	28	<0.05	L supramarginal cx (BA 40)

	60	8	22	26	<0.05	L Broca area (BA 44)
**M1_IE_Mid > M1_Hand**	-38	-18	42	567	<0.001	L M1 (BA 4),L S1(BA 1)
	38	-16	36	517	<0.001	R M1 (BA 4),R PM (BA 6)
	-52	-2	20	283	<0.001	L PM (BA 6)
	56	0	18	148	<0.001	R PM (BA 6)
	16	-64	18	99	<0.001	precuneus
	16	-30	60	77	<0.001	R M1 (BA 4)
	36	6	8	72	<0.001	R Insula (BA 13)
	-34	4	6	54	<0.01	L Insula (BA 13)
	6	10	36	48	<0.01	R ACC (BA 32)
	18	-50	0	45	<0.01	R Occipital cx (BA 18)
	12	-30	48	44	<0.01	R sensory associative cx (BA 5)
	-4	2	60	43	<0.01	SMA (BA 6)
	8	-44	54	40	<0.01	R PCC (BA 31)
	-8	8	38	40	<0.01	L ACC (BA 32)
	-10	-60	-18	38	<0.01	L cerebellum (V-VI)
	12	-14	42	33	<0.01	R ACC (BA 24)
	-30	42	20	33	<0.01	L AntPFC(BA 10)
	38	-10	12	27	<0.05	R Insula (BA 13)
	-4	-6	66	21	<0.05	SMA (BA 6)
	-10	-14	42	21	<0.05	L ACC (BA 24)
	56	-14	8	20	<0.05	R primary auditory cx (BA 41)
	-38	2	-8	20	<0.05	L Insula (BA 13)
**M1_Hand > M1_IE_Mid**	-36	-24	56	1005	<0.001	L M1 (BA 4),L S1(BA 1),L PM (BA 6)
	2	-60	42	84	<0.001	precuneus
	46	-50	28	53	<0.001	R angular cx(BA 39)
	14	46	50	36	<0.01	R FEF (BA 8)
	-40	-54	34	26	<0.05	L angular cx(BA 39)
	22	-52	-52	26	<0.05	R cerebellum (VIII)
	20	-44	-24	22	<0.05	R cerebellum (VI)
	20	56	36	20	<0.05	R DLPFC (BA 9)
**M1_IE_Mid > M1_Foot**	-38	-18	42	2764	<0.001	L S1 (BA 1), L PM (BA 6), L M1 (BA 4)

	38	-16	38	1912	<0.001	R PM (BA 6), R M1 (BA 4), R S1 (BA 1)
	42	-10	16	150	<0.001	R S1 (BA 1)
	-42	-14	16	145	<0.001	L S1 (BA 1)
	20	-52	-24	114	<0.001	R cerebellum (VI)
	-4	-4	14	50	<0.01	L thalamus (Mediodorsal nucleus)
	18	-28	60	40	<0.05	R M1 (BA 4)
	-10	-16	36	28	<0.05	L PCC (BA 23)
**M1_Foot > M1_ IE_Mid**	-4	-42	74	2153	<0.001	precuneous, B SPL (BA 7), B PM (BA 6)
	-16	-38	-54	55	<0.01	L cerebellum (VIII)
	28	-38	-52	35	<0.05	R cerebellum (VIII)
	4	-94	20	28	<0.05	R secondary visual area (BA 18)
**M1_ IE_Mid > M1_Mouth**	-36	-18	42	907	<0.001	L M1 (BA 4), L PM (BA 6), L S1 (BA 1)
	-12	-14	42	759	<0.001	B ACC (BA 32, BA 24), SMA (BA 6), B PM (BA 6), B PCC (BA 31),
	46	-8	50	145	<0.001	R PM (BA 6)
	20	-28	58	136	<0.001	R M1 (BA 4)
	38	14	6	97	<0.001	R Broca area (BA 44), R insula (BA 13)
	14	-42	52	79	<0.001	precuneous
	-34	4	8	45	<0.01	L insula (BA 13)
	12	-6	72	39	<0.01	R PM (BA 6)
	-6	-10	68	37	<0.05	SMA (BA 6)
	6	-64	-18	35	<0.05	cerebellum vermis (VI)
**M1_Mouth > M1_ IE_Mid**	-48	-14	36	982	<0.001	L M1 (BA 4), L PM (BA 6), L S1 (BA 1)
	54	-6	32	777	<0.001	R S1 (BA 1), R M1 (BA 4)
	-40	8	54	26	<0.05	L PM (BA 6)
**M1_ IE_Mid > SMA**	-38	-18	42	1229	<0.001	B M1 (BA 4)
	36	-18	38	1048	<0.001	B PM (BA 6), SMA (BA 6)
	30	-76	-40	232	<0.001	R SPL (BA 7)
	28	-50	-58	59	<0.001	R cerebellum (VIII)
	-48	32	-2	56	<0.001	L Broca area (BA 45)
	18	-86	-20	55	<0.001	L cerebellum (CRUS I)
	-36	-82	-36	48	<0.01	L cerebellum (CRUS II)
	-40	16	30	35	<0.05	L Broca area (BA 44)

	6	-60	16	29	<0.05	R PCC (BA 31)
**SMA > M1_ IE_Mid**	0	-8	54	1876	<0.001	B ACC (BA 32), B PM (BA 6), SMA (BA 6), L SPL (BA 7), precuneous, PCC (BA 31)
	64	-24	30	443	<0.001	R supramarginal cx (BA 40)
	42	2	14	213	<0.001	R PM (BA 6)
	-50	-30	22	198	<0.001	L supramarginal cx (BA 40)
	-48	0	6	160	<0.001	L PM (BA 6)
	26	-46	70	115	<0.001	R SPL (BA 7)
	50	20	-4	87	<0.001	R pars orbitalis (BA 47)
	-40	-2	-2	86	<0.001	L insula (BA 13)
	-30	-50	-56	62	<0.001	L cerebellum (VIII)
	42	-38	68	28	<0.05	R S1 (BA 1)
	-36	20	10	27	<0.05	L Broca area (BA 45)
	38	-14	-2	26	<0.05	R insula (BA 13)
**M1_IE_Inf > M1_Foot**	62	2	14	3925	<0.001	RM1 (BA 4), R PM (BA 6), R S1 (BA 1), R insula (BA 13), R A1 (BA 41), R supramarginal cx (BA 40), R sup temporal cx (BA 22)
	-58	-2	16	3630	<0.001	L M1 (BA 4), L PM (BA 6), L S1 (BA 1), L insula (BA 13), L A1 (BA 41), L supramarginal cx (BA 40), L sup temporal cx (BA 22)
	-4	-4	56	90	<0.001	SMA (BA 6)
	-14	-60	-16	87	<0.001	L cerebellum (IV-V-VI)
	12	-64	-48	61	<0.01	R cerebellum (VIII-IX)
	4	-6	16	37	<0.05	R thalamus (Mediodorsal nucleus)
	44	4	-16	29	<0.05	R temporal cx (BA 22)
	16	-64	-22	28	<0.05	R cerebellum (VI)
**M1_Foot > M1_IE_Inf**	-4	-42	72	2233	<0.001	R SPL (BA 7), B S1 (BA 1), B M1 (BA 4), precuneous
	-36	-74	26	61	<0.01	L secondary visual cx (BA 19)
	-14	-42	-58	48	<0.01	L cerebellum (VIII-IX)
	16	-46	-56	37	<0.05	R cerebellum (VIII-IX)
	-48	-62	-44	60	<0.05	L cerebellum (CRUS II)
**M1_IE_Inf > M1_Hand**	-54	-4	14	2205	<0.001	L PM (BA 6), L S1 (BA 1), L insula (BA 13), L Broca area (BA 44), L A1 (BA 41), L temporal cx (BA 22)
	62	2	14	2187	<0.001	R PM (BA 6), R S1 (BA 1), R insula (BA 13), R Broca area (BA 44), R A1 (BA 41), R temporal cx (BA 22), R supramarginal cx (BA 40)
	-16	-60	-16	175	<0.001	L cerebellum (VI)

	-4	14	34	148	<0.001	B ACC (BA 32)
	64	-26	36	143	<0.001	R supramarginal cx (BA 40)
	4	4	54	119	<0.001	SMA (BA 6)
	-56	-36	50	118	<0.001	L supramarginal cx (BA 40)
	-34	36	16	100	<0.001	L AntPFC (BA 10)
	16	-64	18	36	<0.05	Precuneous cx
	-42	34	22	30	<0.05	L DLPFC (BA 46)
**M1_Hand > M1_IE_Inf**	-34	-26	56	1626	<0.001	L M1 (BA 4), L PM (BA 6)
	-6	-46	42	144	<0.001	Precuneous cx, PCC (BA 31)
	22	-46	-26	79	<0.001	R cerebellum (IV-V-VI)
	36	-22	54	55	<0.01	R M1 (BA 4)
	16	-86	-38	49	<0.01	R cerebellum (CRUS II)
	-44	-54	32	25	<0.05	L angular cx(BA 39)
**M1_IE_Inf > M1_Mouth**	-56	-2	12	1611	<0.001	L insula (BA 13), L PM (BA 6)
	64	6	4	1167	<0.001	R PM (BA 6), R Insula (BA 13), R Broca's area (BA 44), R primary auditory cx (BA 41), R sup temporal cx(BA 22)
	-10	4	38	620	<0.001	ACC (BA 32), SMA (BA 6)
	62	-30	34	335	<0.001	R supramarginal cx(BA 40)
	-46	-38	20	131	<0.001	L sup temporal cx(BA 22)
	36	46	36	52	<0.01	R DLPFC (BA 9)
	-20	-2	18	43	<0.01	L caudate
	44	-2	46	30	<0.05	R PM (BA 6)
**M1_Mouth > M1_IE_Inf**	-48	-14	36	709	<0.001	L S1 (BA 1), L M1 (BA 4)
	48	-10	34	409	<0.001	R M1 (BA 4)
	-12	44	40	112	<0.001	L FEF (BA 8)
	44	-70	-40	75	<0.001	R cerebellum (CRUS I)
	-44	-52	36	43	<0.01	L angular cx(BA 39)
	-4	-48	36	32	<0.05	L PCC (BA 23)
	-40	-34	60	28	<0.05	L S1 (BA 1)
	-8	32	56	24	<0.05	L FEF (BA 8)
**M1_IE_Inf > SMA**	-56	-6	14	1571	<0.001	L PM (BA 6), L M1 (BA 4), L S1(BA 1), L Broca area (BA 44), L sup temporal cx (BA 22)

	60	-2	18	1506	<0.001	R PM (BA 6),R M1 (BA 4), R S1(BA 1), R Broca area (BA 44), R sup temporal cx (BA 22)
	36	-10	14	59	<0.01	R insula cx (BA 13)
	-36	-10	12	57	<0.01	L insula cx (BA 13)
	-48	34	2	44	<0.01	L pars triangularis (BA 45)
	-14	-86	-8	40	<0.01	L secondary visual area (BA 18)
	-14	-60	-16	40	<0.01	L cerebellum (VI)
	-26	-66	-14	37	<0.05	L secondary visual area (BA 19)
	-44	14	20	34	<0.05	L Broca area (BA 44)
	-60	-42	10	34	<0.05	L supramarginal cx (BA 40)
	-64	-26	6	33	<0.05	L mid temporal cx (BA 21)
	-44	-36	14	32	<0.05	L sup temporal cx (BA 22)
	64	-24	-18	25	<0.05	R mid temporal cx (BA 21)
	12	-64	-42	22	<0.05	R cerebellum (VIII)
**SMA > M1_IE_Inf**	0	-6	54	2726	<0.001	B PM (BA 6), SMA (BA 6), B SPL (BA 7), B S1 (BA 1), B ACC (BA 32), precuneous
	16	-16	72	143	<0.001	R PM (BA 6)
	22	-52	-54	80	<0.001	R cerebellum (VIII)
	-42	-24	20	44	<0.01	L supramarginal cx (BA 40)
	14	-44	56	29	<0.05	R SPL (BA 7)
	34	-38	64	27	<0.05	R S1 (BA 1)
	6	18	34	26	<0.05	B ACC (BA 32)
	36	22	-10	26	<0.05	R pars orbitalis (BA 47)
	30	-36	-30	24	<0.05	R cerebellum (IV-V-VI)
**M1_IE_Mid > M1_IE_Sup**	-38	-18	42	1781	<0.001	L PM (BA 6), L S1 (BA 1)
	48	-8	44	1423	<0.001	R PM (BA 6), R M1 (BA 4), R S1 (BA 1),
	16	-54	-22	81	<0.001	R cerebellum (IV-V-VI)
	-34	-8	12	34	<0.05	L M1 (BA 4)
	-38	0	42	28	<0.01	L PM (BA 6)
**M1_IE_Sup > M1_IE_Mid**	-22	-36	58	2984	<0.001	B S1 (BA 1), B M1 (BA 4), B PM (BA 6), SMA (BA 6), B PCC (BA 31), B SPL (BA 7), L Secondary sensory cx (BA 5), precuneous
	46	-26	26	40	<0.01	R supramarginal cx (BA 40)
	32	-34	-34	38	<0.05	R cerebellum (IV-V-VI)

	8	-4	44	35	<0.05	B ACC (BA 32), SMA (BA 6)
	-18	-46	-52	32	<0.05	L cerebellum (IX)
	34	-22	22	25	<0.05	R S1 (BA 1)
	-8	-16	78	24	<0.05	L PM (BA 6), SMA (BA 6)
**M1_IE_Inf > M1_IE_Sup**	-54	-8	14	2955	<0.001	L M1 (BA 4), L S1 (BA 1), L PM cx (BA 6), L supramarginal cx (BA 40), L sup temporal cx (BA 22), L A1 (BA 41)
	62	2	14	2885	<0.001	R M1 (BA 4), R S1 (BA 1), R PM cx (BA 6), R supramarginal cx (BA 40), R sup temporal cx (BA 22), R A1 (BA 41)
	-18	-66	-18	192	<0.001	L cerebellum (VI)
	22	-66	-20	109	<0.001	R cerebellum (VI)
	-60	-22	46	68	<0.001	L supramarginal cx(BA 40)
	-6	4	52	40	<0.01	SMA (BA 6)
	12	-62	-50	36	<0.05	R cerebellum (IV-V-VI)
	46	-42	18	32	<0.05	R supramarginal cx (BA 40)
	-18	4	14	32	<0.05	L caudate
	46	-26	-2	26	<0.05	R superior temporal cx (BA 22)
**M1_IE_Sup > M1_IE_Inf**	-20	-34	62	3884	<0.001	B S1 (BA 1), B M1 (BA 4), B PM (BA 6), SMA (BA 6), B ACC (BA 32), L associative sensory cx (BA 5)
	26	-36	-28	39	<0.01	R cerebellum (VIII)
**M1_IE_Inf > M1_IE_Mid**	-54	-8	12	1663	<0.001	L PM (BA 6), L S1 (BA 1), L insula (BA 13), L sup temporal cx (BA 22), L A1 (BA 41)
	40	4	-6	720	<0.001	R PM (BA 6), R S1 (BA 1), R insula (BA 13), R sup temporal cx (BA 22),R A1 (BA 41)
	60	-14	26	294	<0.001	R supramarginal cx(BA 40)
	-44	-38	18	36	<0.01	L supramarginal cx(BA 40)
**M1_IE_Mid > M1_IE_Inf**	-38	-18	44	889	<0.001	L M1 (BA4), L S1 (BA 1)
	38	-16	42	144	<0.001	R M 1 (BA 4)
	6	58	10	26	<0.05	R ALPFC (BA 10)
	16	-30	62	24	<0.05	R M1 (BA 4)
**M1_Hand > M1_Foot**	-36	-24	60	2163	<0.001	L S1 (BA 1), L M1 (BA 4), L PM cx (BA 6)
	56	-12	46	543	<0.001	R PM cx (BA 6)
	20	-48	-26	226	<0.001	R cerebellum (IV-V-VI)
	-56	-2	34	83	<0.001	L PM cx (BA 6)
	-46	-18	20	64	<0.001	L S1 (BA 1)

	12	-58	-42	57	<0.01	R PM cx (BA 6)
	-8	-10	54	49	<0.01	SMA (BA 6)
**M1_Foot > M1_Hand**	-4	-42	72	2685	<0.001	precuneous, B sensory associative cx (BA 5), B M1 (BA 4), B SPL (BA 7), R S1 (BA 1)
	-14	-40	-56	85	<0.001	L cerebellum (VIII-IX)
	-36	46	24	47	<0.001	L ALPFC (BA 10)
	18	-70	34	38	<0.05	precuneous
	26	-28	-30	37	<0.05	R cerebellum (IV-V)
	4	-90	32	35	<0.05	R secondary visual cx (BA 19)
	28	40	20	26	<0.05	R AntPFC (BA 10)
**M1_Hand > M1_Mouth**	-36	-24	58	1351	<0.001	L PM cx (BA 6), L M1 (BA 4)
	-8	-8	50	190	<0.001	SMA (BA 6)
	20	-44	-24	89	<0.001	R cerebellum (IV-V)
	22	-54	-54	32	<0.01	R cerebellum (VIII)
	56	-52	26	24	<0.05	R angular cx (BA 39)
	28	-20	50	21	<0.05	R M1 (BA 4)
**M1_Mouth > M1_Hand**	-46	-14	36	1141	<0.001	L PM cx (BA 6), L M1 (BA 4)
	54	-6	34	1082	<0.001	R PM cx (BA 6), R S1 (BA 1), R M1 (BA 4)
	38	-4	8	81	<0.001	R insula (BA 13)
	-14	-66	-18	76	<0.001	L cerebellum (VI)
	-46	-52	58	58	<0.001	L supramarginal cx(BA 40)
	-36	-6	10	46	<0.01	L insula (BA 13)
	52	-36	58	43	<0.01	R supramarginal cx(BA 40)
	-42	10	54	21	<0.05	L PM cx (BA 6)
	-42	34	24	20	<0.05	R DLPFC (BA 9)
**M1_Foot > M1_Mouth**	-4	-42	72	2558	<0.001	precuneous, B SPL (BA 7), B SMA (BA 6), PCC (BA 31),
	16	-44	-52	63	<0.01	R cerebellum (VI)
	-14	-42	-60	62	<0.01	L cerebellum (VIII-IX)
	4	4	46	50	<0.01	B ACC (BA 32), SMA (BA 6)
	24	-34	-26	48	<0.01	R cerebellum (IV-V)
	0	-96	8	47	<0.01	R secondary visual cx (BA 18)
	36	46	36	43	<0.01	R DLPFC (BA 9)

**M1_Mouth > M1_Foot**	-48	-14	36	2565	<0.001	L S1 (BA 1), L M1 (BA 4), L insula (BA 13), L A1 (BA 41)
	62	-6	32	2453	<0.001	R M1 (BA 4), R S1 (BA 1), R PM cx (BA 6), R supramarginal cx (BA 40)
	20	-54	-20	82	<0.001	R cerebellum (IV-V)
	-14	-58	-18	57	<0.01	L cerebellum (VI)
	28	-12	-16	26	<0.05	R hippocampus
**M1_Foot > SMA**	-4	-40	74	1215	<0.001	precuneous, B S1 (BA 1), B SPL (BA 7)
	20	-72	-34	132	<0.001	R cerebellum (CRUS I-II)
	6	-88	30	99	<0.001	R secondary visual area (BA 18)
	-36	-74	26	93	<0.001	L angular cx (BA 39)
	-46	-64	-44	77	<0.001	L cerebellum (CRUS II)
	-32	30	52	68	<0.01	L FEF (BA 8)
	48	-70	-16	60	<0.01	R cerebellum (CRUS I)
	36	-64	-48	53	<0.01	R cerebellum (VIII- IX)
	-4	-48	44	48	<0.01	precuneous
	-22	-78	-38	45	<0.01	L cerebellum (CRUS I-II)
	-56	-56	-6	38	<0.05	L fusiform cx (BA 37)
	-52	-72	-14	35	<0.05	L secondary visual area (BA 19)
	6	62	32	31	<0.05	R DLPFC (BA 9)
	-52	42	2	31	<0.05	R DLPFC (BA 46)
	-10	-40	-50	31	<0.05	L cerebellum (IX)
**SMA > M1_Foot**	-2	-6	54	4047	<0.001	B PM (BA 6), B FEF (BA 8), B ACC (BA 32), SMA (BA 6), B S1 (BA 1), B PCC (BA 31)
	40	2	12	2156	<0.001	R insula (BA 13), R, M1 (BA 4), R PM (BA 6), R putamen, R pallidum, R broca area (BA 44), R pars orbitalis (BA 47)
	-38	-4	14	1555	<0.001	L insula (BA 13), L M1 (BA 4), L PM (BA 6), L putamen, L pallidum, L broca area (BA 44), L pars orbitalis (BA 47)
	42	-38	66	576	<0.001	R S1 (BA 1), R supramarginal cx (BA 40)
	22	-52	-22	99	<0.001	R cerebellum (IV-V-VI)
	2	-6	16	62	<0.01	R thalamus (Mediodorsal nucleus)
	-58	2	34	57	<0.01	L cerebellum (VII-VIII, CRUS II)
	24	-54	-52	51	<0.01	R cerebellum (VIII-IX)
	56	-16	44	49	<0.01	R S1 (BA 1)
	12	-50	-18	40	<0.05	R cerebellum (IV-V)

	-56	-24	44	37	<0.05	R S1 (BA 1)
**M1_Hand > SMA**	-36	-24	56	1112	<0.001	L M1 (BA 4)
	38	-84	-32	193	<0.001	R cerebellum (CRUS I-II)
	-50	-54	34	154	<0.001	L angular cx (BA 39)
	-20	22	46	107	<0.001	R S1 (BA 1), R SPL (BA 7)
	-24	-88	-34	97	<0.001	L cerebellum (CRUS I-II)
	16	46	48	87	<0.001	R FEF (BA 8)
	-56	-10	32	84	<0.001	L M1 (BA 4), L S1 (BA 1)
	54	-52	26	83	<0.001	R angular cx (BA 39)
	40	-68	-40	62	<0.001	R cerebellum (CRUS I-II)
	62	-14	-12	49	<0.01	R sup temporal cx (BA 22)
	66	-2	22	43	<0.01	R M1 (BA 4), R PM (BA 6)
	-12	-82	-40	95	<0.01	L cerebellum (CRUS II)
	-38	16	30	22	<0.05	L DLPFC (BA 9)
**SMA > M1_Hand**	0	-4	54	3831	<0.001	B ACC (BA 32), SMA (BA 6), B PM (BA 6), R FEF (BA 8), precuneous, B PCC (BA 31), L SPL (BA 7)
	38	6	10	1483	<0.001	R insula (BA 13), R PM (BA 6), R putamen, R pallidum, R broca area (BA 44), R pars orbitalis (BA 47)
	-36	4	6	976	<0.001	L insula (BA 13), L PM (BA 6), L putamen, L broca area (BA 44), L pars orbitalis (BA 47)
	56	-28	32	856	<0.001	R supramarginal cx (BA 40), R S1 (BA 1), R insula (BA 13)
	-56	-34	36	284	<0.001	L supramarginal cx (BA 40), L S1 (BA 1)
	28	-46	72	137	<0.001	R SPL (BA 7), R S1 (BA 1)
	-30	38	20	102	<0.001	L AntPFC (BA 10)
	32	34	30	94	<0.001	R DLPFC (BA 9)
	-28	-48	-28	79	<0.001	L cerebellum (IV-V-VI)
	32	-42	44	33	<0.05	R cerebellum (CRUS I)
	-30	-50	-56	32	<0.05	L cerebellum (VIII)
	54	-32	58	30	<0.05	R supramarginal cx (BA 40)
	36	-48	-26	26	<0.05	R cerebellum (VI)
	-62	-16	14	23	<0.05	L supramarginal cx (BA 40)
**M1_Mouth > SMA**	-48	-14	36	1258	<0.001	L M1 (BA 4), L S1 (BA 1), L PM (BA 6)
	54	-6	34	1063	<0.001	R M1 (BA 4), R S1 (BA 1), R PM (BA 6)

	60	-28	32	663	<0.001	R supramarginal cx (BA 40)
	32	-66	-36	312	<0.001	R cerebellum (VII-VIII, CRUS I-II)
	-44	6	50	244	<0.001	R FEF (BA 8)
	-50	-54	34	204	<0.001	L angular cx (BA 39)
	-42	-70	-32	144	<0.001	L cerebellum (CRUS I-II)
	-48	34	-2	113	<0.001	L pars orbitalis (BA 47), L pars triangularis (BA 45)
	-10	18	62	93	<0.001	L FEF (BA 8)
	-22	50	36	36	<0.01	L DLPFC (BA 9)
	-30	-86	-30	36	<0.01	L cerebellum (CRUS I-II)
	38	-60	40	29	<0.05	R angular cx (BA 39)
	20	-72	54	28	<0.05	R SPL (BA 7)
	18	-58	-22	27	<0.05	R cerebellum (VI)
	64	-24	-18	26	<0.05	R middle temporal cx (BA 21)
	16	-84	-22	23	<0.05	R cerebellum (CRUS I)
	-12	-74	60	20	<0.05	L SPL (BA 7)
	16	-82	-38	19	<0.05	R cerebellum (CRUS II)
**SMA > M1_Mouth**	0	-8	54	4723	<0.001	B ACC (BA 32), SMA (BA 6), B PM (BA 6), R FEF (BA 8), precuneous, B PCC (BA 31), L SPL (BA 7)
	36	14	8	929	<0.001	R insula (BA 13), R PM (BA 6), R putamen, broca area (BA 44)
	-38	-16	4	790	<0.001	L insula (BA 13), L PM (BA 6), L putamen, L broca area (BA 44)
	-48	-30	22	237	<0.001	L supramarginal cx (BA 40), L S1 (BA 1)
	38	-14	-2	174	<0.001	R insula (BA 13), R putamen
	32	-42	-50	113	<0.001	R cerebellum (VIII-IX)
	-28	-50	-56	97	<0.001	L cerebellum (VIII-IX)
	32	44	32	92	<0.001	R DLPFC (BA 9)
	24	-38	68	51	<0.01	R S1 (BA 1), R SPL (BA 7)
	22	-38	-26	27	<0.05	R cerebellum (IV-V)
	-38	-8	54	25	<0.05	L PM (BA 6)
	-28	36	20	20	<0.05	L AntPFC (BA 10)
	28	-44	-28	20	<0.05	R cerebellum (VI)

Note: ***cluster forming threshold p<.001, cluster-level p-FDR<.05.*** *R: right, L: left, B: bilateral, BA: Brodmann area, cx: cortex, AntPFC: anterior prefrontal cortex, S1: primary sensory cortex, M1: primary motor cortex, PM : premotor cortex, SMA: supplementary motor area, FEF: frontal eye field, ACC: anterior cingulate cortex, PCC: posterior cingulate cortex, DLPFC: dorsolateral prefrontal cortex, SPL: superior parietal cortex, A1: primary auditory cortex, M1_Inf_IE: inferior intereffector, M1_Mid_IE: middle intereffector, M1_Sup_IE: superior intereffector.*

SI Appendix C: Cross-validation

SI Appendix C1: Cross-validation of the seed-to-voxels connectivity

This table reports the DSC for each seed across all the subsets for the whole group and the PS vs non-PS comparisons

	Whole group DSC (mean ±SD across the 10 subsets)	PS vs non-PS DSC (mean ±SD across the 20 subsets)
**M1_Sup_IE**	0.94±0.02	0.87±0.07
**M1_Mid_IE**	0.84±0.02	0.85±0.09
**M1_Inf_IE**	0.81±0.03	/
**M1_Foot**	0.90±0.04	/
**M1_Hand**	0.92±0.02	/
**M1_Mouth**	0.79±0.06	/
**SMA**	0.93±0.02	/

DSC: Dice Similarity Coefficient, PS: patients with psychomotor slowing, non-PS: patients without psychomotor slowing, M1_Inf_IE: inferior intereffector, M1_Mid_IE: middle intereffector, M1_Sup_IE: superior intereffector, SMA: supplementary motor area, M1: primary motor area.

SI Appendix D: Complementary data on between-group comparisons of the seed-to-voxels rs-FC with M1 effectors and intereffectors

SI Appendix D1: Between-groups difference in effectors and intereffectors seed-to-voxel rs-FC

	x	y	z	size	p-FDR	Brain Areas
**M1_Foot**						
**HC > PS**	-34	-60	46	170	<0.01	L angular cx(BA 39)
	-54	-56	-8	147	<0.01	L fusiform cx (BA 37)
	-48	18	32	110	<0.05	L FEF (BA 8)
	-44	-46	54	95	<0.05	L supramarginal cx(BA 40)
	-34	-44	-44	83	<0.05	R cerebellum (BA VIII)
**PS > HC**	12	-84	8	109	<0.05	R visual cx (BA 17)
**M1_Hand**						
**HC > PS**	42	-60	-2	371	<0.001	R fusiform cx (BA 37)
	62	-10	30	332	<0.001	R M1 (BA 4), R S1 (BA 1), R PM (BA 6)
	34	-32	54	301	<0.001	R S1 (BA 1)
	-50	-26	48	259	<0.001	L M1 (BA 4), L S1 (BA 1)
	-6	54	32	152	<0.001	L DLPFC (BA 9)
	-48	-74	0	133	<0.001	L secondary visual cx (BA 19)
	56	-58	28	65	<0.05	R angular cx(BA 39)
**PS > HC**	-12	-68	6	2554	<0.001	B primary visual cx (BA 17), B secondary visual cx (BA 18), B cerebellum (IV-V-VI), cerebellum (vermis IV-V-VI), precuneus
	30	42	34	746	<0.001	R anterior prefrontal cx (BA 10), R DLPFC (BA 9)
	52	-36	38	433	<0.001	R supramarginal cx(BA 40), R angular cx(BA 39)
	2	20	42	377	<0.001	SMA (BA 6), ACC (BA 32), R FEF (BA 8)
	-32	-50	-28	340	<0.001	L cerebellum (VI, CRUS I)
	-28	46	34	335	<0.001	L anterior prefrontal cx (BA 10), L DLPFC (BA 9)
	42	18	0	329	<0.001	R Broca area (BA 44-45), R insula (BA 13)
	-34	14	8	259	<0.001	L Broca area (BA 44-45), R insula (BA 13)
	2	-28	30	243	<0.001	PCC (BA 31)

	-10	-62	36	178	<0.001	precuneus
	26	-54	-26	171	<0.001	R cerebellum (VI, CRUS I)
	16	-62	40	118	<0.01	precuneus
	-38	50	20	78	<0.05	L anterior prefrontal cx (BA 10)
	4	-38	44	69	<0.05	PCC (BA 31)
	0	-80	44	56	<0.05	precuneus
**M1_Mouth**						
**HC > PS**	42	-60	-2	233	<0.001	L secondary visual cx (BA 19)
	54	-18	52	110	<0.01	R S1 (BA 1)
**PS > HC**	-10	-62	36	1224	<0.001	precuneus, PCC (BA 31)
	60	-46	42	264	<0.001	R angular cx(BA 39), R supramarginal cx(BA 40)
	-48	-48	40	221	<0.001	L angular cx(BA 39), L supramarginal cx(BA 40)
	-10	-16	12	107	<0.01	L thalamus (centromedian nucleus)
	-32	-40	-34	99	<0.01	L cerebellum (VI)
	6	-42	22	83	<0.05	precuneus, PCC (BA 31)
	-36	38	42	72	<0.05	L DLPFC (BA 9)
**HC > non-PS**	54	-66	4	199	<0.001	R fusiform cx (BA 37)
**non-PS > HC**	8	-32	34	170	<0.001	R PCC (BA 23)
**SMA**						
**HC > PS**						
**PS > HC**	0	-70	8	2683	<0.001	B primary and secondary visual cx (BA 17-18), precuneus, cerebellum IV-V-VI
	-20	-28	4	97	<0.05	L thalamus (pulvinar nucleus)
**HC > non-PS**						
**non-PS > HC**	0	-64	8	123	<0.05	B secondary visual cx (BA 18)
	10	-64	60	86	<0.05	R SPL (BA 7)
**M1_IE_Sup**						
**HC > PS**	70	-6	22	155	<0.01	R M1 (BA 4)
	58	-24	6	96	<0.05	R primary auditory cx (BA 41
**PS > HC**	6	-68	10	2489	<0.001	B primary and secondary visual cx (BA 17-18), precuneus, cerebellum IV-V-VI
	40	40	8	156	<0.01	R DLPFC (BA 46)
	-10	-40	-52	82	<0.05	L cerebellum (BA IX)

	-42	-50	-56	78	<0.05	L cerebellum (BA VIII)
**HC > non-PS**	-22	-30	50	464	<0.001	L SPL (BA 7), L sensory associative area (BA 5)
	10	-16	34	138	<0.01	PCC (BA 23)
	-8	0	34	123	<0.01	ACC (BA 24)
	-46	-78	-2	92	<0.05	L secondary visual cx (BA 19)
	-40	-30	58	84	<0.05	L S1 (BA 1)
	-32	-2	-4	81	<0.05	L putamen
	20	-36	56	66	<0.05	R M1 (BA 4)
**non-PS > HC**	28	-26	74	195	<0.001	R M1 (BA 4), R PM (BA 6)
	-6	-48	-2	68	<0.05	L cerebellum (IV-V)
**PS > non-PS**	0	-94	8	88	<0.05	B primary and secondary visual cx (BA 17-18)
**M1_IE_Mid**						
**HC > PS**	50	-68	-10	187	<0.001	R fusiform (BA 37)
	14	-34	18	72	<0.05	R thalamus (ventral lateral nucleus)
**PS > HC**	4	-68	8	1503	<0.001	B primary and secondary visual cx (BA 17-18)
	14	-62	36	463	<0.001	precuneus
	-22	-58	-48	229	<0.001	L cerebellum (BA VIII)
	32	42	32	152	<0.001	R DLPFC (BA 9)
	2	20	40	96	<0.01	ACC (BA 32)
	-34	34	34	88	<0.05	L DLPFC (BA 9)
	-34	14	6	80	<0.05	L insula (BA 13)
	14	-58	-12	68	<0.05	R cerebellum (IV-V-VI)
	-8	-14	10	114	<0.01	L thalamus (centromedian nucleus)
**non-PS > HC**	40	-24	24	79	<0.001	R S1 (BA 1)
**non-PS > PS**	36	-42	68	80	<0.05	R S1 (BA 1), R angular cx (BA 39)
	22	34	50	67	<0.05	R FEF (BA 8)
**PS > non-PS**	-04	-78	-4	872	<0.001	B primary and secondary visual cx (BA 17-18)
	-32	-20	38	120	<0.05	L S1 (BA 1), L M1 (BA 4), L PM (BA 6)
**M1_IE_Inf**						
**HC > PS**	46	-68	-8	88	<0.05	R fusiform cx (BA 37)
	-36	-84	-24	76	<0.05	L cerebellum (CRUS I)
**PS > HC**	4	-68	8	545	<0.001	B primary and secondary visual cx (BA 17-18)
	-24	-58	-48	145	<0.01	L cerebellum (BA VIII)

	-6	-14	10	118	<0.01	L thalamus (centromedian nucleus)
**non-PS > HC**	-34	-30	20	174	<0.001	L S1 (BA 1)
	-8	-20	8	149	<0.01	L thalamus (centromedian nucleus)
	6	2	74	139	<0.01	SMA (BA 6)
	36	-24	24	132	<0.01	R supramarginal cx(BA 40)

Note: R: right, L: left, B: bilateral, BA: Brodmann area, cx: cortex, AntPFC: anterior prefrontal cortex, S1: primary sensory cortex, M1: primary motor cortex, PM : premotor cortex, SMA: supplementary motor area, FEF: frontal eye field, ACC: anterior cingulate cortex, PCC: posterior cingulate cortex, DLPFC: dorsolateral prefrontal cortex, SPL: superior parietal cortex, A1: primary auditory cortex, PS: patients with psychomotor slowing, non-PS: patients without psychomotor slowing, HC: healthy controls, M1_Inf_IE: inferior intereffector, M1_Mid_IE: middle intereffector, M1_Sup_IE: superior intereffector.

SI Appendix D2: Differences in seed-to-voxels resting-state functional connectivity between patients without psychomotor slowing and healthy controls.

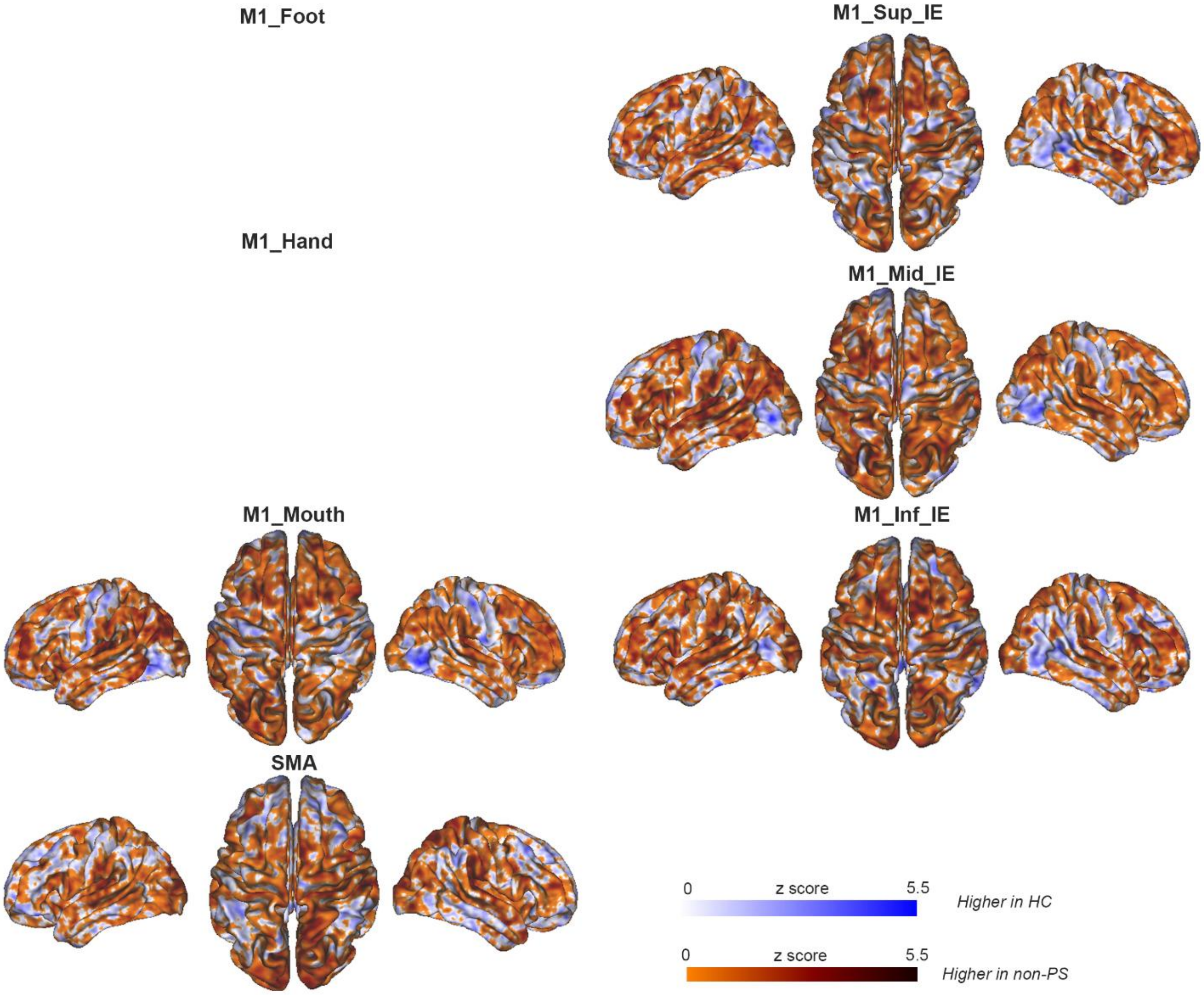

Note: Seed-to-voxels resting-state functional connectivity (rs-FC) between patients without psychomotor slowing and healthy controls. The maps are unthresholded and corrected for age, sex, and framewise displacement as covariates (For thresholded results, see SI Appendix D1). Each of the three M1 effectors (M1_Foot (x=-4, y=-40, z=70), M1_Hand (x=-35, y=-24, z=56), M1_Mouth (x=-46, y=-15, z=34)), three M1 intereffectors (M1_Sup_IE (x=-19, y=-34, z=59), M1_Mid_IE (x=-38, y=-18, z=44), M1_Inf_IE (x=-54, y=-3, z=14)); and SMA (x=-5, y=-7, z=52)) were used as seeds.

The orange scale reflects higher rs-FC in patients without psychomotor slowing compared to healthy controls, while the blue scale reflects higher rs-FC in healthy controls compared to patients without psychomotor slowing. Darker areas reflect stronger rs-FC. No between-group differences were observed with M1_Foot or M1_Hand as seeds.

Abbreviations: *M1_Inf_IE: inferior intereffector, M1_Mid_IE: middle intereffector, M1_Sup_IE: superior intereffector, SMA: supplementary motor area, M1: primary motor cortex, z: axial MNI coordinate.*

Supplementary E: Complementary data on the effectors and intereffectors seed-to-voxel rs-FC association with motor behavior

SI Appendix E1: Cluster statistics related to effectors and intereffectors seed-to-voxels rs-FC association with measured activity level

	x	y	z	size	p-FDR	Brain Areas
**M1_Foot**						
**HC**						
**positive association with actigraphy**	24	-84	0	190	<0.001	R primary and secondary visual (BA 17-18)
	8	-96	16	178	<0.001	R primary and secondary visual (BA 17-18)
	-2	-72	6	110	<0.01	L primary and secondary visual (BA 17-18), precuneus
**M1_Hand**						
**PS**						
**positive association with actigraphy**	-54	-32	48	286	<0.001	L supramarginal cx(BA 40), L S1 (BA 1)
**M1_Mouth**						
**non-PS**						
**negative association with actigraphy**	-4	-20	74	82	<0.05	SMA (BA 6)
	0	-86	-34	70	<0.05	L cerebellum (CRUS II)
**M1_IE_Sup**						
**non-PS**						
**negative association with actigraphy**	-46	10	28	126	<0.001	L PM (BA 6)
	0	2	54	58	<0.05	SMA (BA 6)
**M1_IE_Mid**						
**PS**						
**positive association with actigraphy**	-54	-18	40	125	<0.01	L S1 (BA 1)
	-42	-18	60	97	<0.05	L M1 (BA 4)
**M1_IE_Inf**						
**PS**						
**positive association with actigraphy**	-30	-50	54	350	<0.001	L primary and secondary visual (BA 17-18), precuneus, L angular cortex (BA 39)

*Note: **cluster forming threshold p<.005, cluster-level p-FDR<.05.** R: right, L: left, B: bilateral, BA: Brodmann area, cx: cortex, S1: primary sensory cortex, M1: primary motor cortex, PM : premotor cortex, SMA: supplementary motor area, PS: patients with psychomotor slowing, non-PS: patients without psychomotor slowing, M1_Inf_IE: inferior intereffector, M1_Mid_IE: middle intereffector, M1_Sup_IE: superior intereffector.*

SI Appendix E2: Cluster statistics related to effectors and intereffectors seed-to-voxels rs-FC association with measured manual dexterity

	x	y	z	size	p-FDR	Brain Areas
**M1_Hand**						
**non-PS**						
**positive association with dexterity**	-16	-28	-04	183	<0.001	B thalamus (Pulvinar nucleus)
**M1_IE_Inf**						
**PS**						
**positive association with dexterity**	14	-56	+64	242	<0.001	R parietal cx (BA 7)
	38	-22	40	175	<0.001	R S1 (BA 1)

Note: ***cluster forming threshold p<.005, cluster-level p-FDR<.05.*** *R: right, L: left, B: bilateral, BA: Brodmann area, cx: cortex, S1: primary sensory cortex, M1: primary motor cortex, PM : premotor cortex, SMA: supplementary motor area, PS: patients with psychomotor slowing, non-PS: patients without psychomotor slowing, M1_Inf_IE: inferior intereffector, M1_Mid_IE: middle intereffector, M1_Sup_IE: superior intereffector.*

SI Appendix E3: Cluster statistics related to effectors and intereffectors seed-to-voxel rs-FC association with SRRS in patients with psychomotor slowing

	x	y	z	size	p-FDR	Brain Areas
**Left M1_Hand**						
**positive association with SRRS**	-16	-86	-34	61	<0.05	L cerebellum (CRUS I-II)
	-32	-68	-54	212	<0.001	L cerebellum (VIII)
	22	-72	-48	120	<0.001	R cerebellum (VIII)
	32	-2	56	126	<0.01	R PM (BA 6)
**Left M1_IE_Sup**						
**positive association with SRRS**	12	-70	-30	90	<0.01	R cerebellum (VI, CRUS I-II)
	2	-60	-16	93	<0.01	Vermis VI
	-18	-70	-30	135	<0.01	L cerebellum (CRUS I-II)
	-20	-58	-50	122	<0.01	L cerebellum (VIII)
**Left M1_IE_Mid**						
**positive association with SRRS**	-14	-66	-42	132	<0.001	L cerebellum (VIII, CRUS II)
**SMA**						
**negative association with SRRS**	54	-28	-8	90	<0.01	R Middle temporal gyrus (BA 21)
	50	8	14	129	<0.01	R Broca area (BA 44)
	30	32	44	85	<0.01	R FEF (BA 8)
	-44	12	8	69	<0.05	L Broca area (BA 44)
**R M1_IE_Inf**						
**negative association with SRRS**	-56	-40	02	150	<0.001	L temporal gyrus (BA 21)
**R M1_Hand**						
**positive association with SRRS**	-20	-68	-50	81	<0.01	L cerebellum (VIII)
	36	0	60	55	<0.05	R PM (BA 6)
	42	34	16	53	<0.05	R DLPFC (BA 46)
	46	08	32	49	<0.05	R Broca area (BA 44)

Note: R: right, L: left, B: bilateral, BA: Brodmann area, cx: cortex, AntPFC: anterior prefrontal cortex, M1: primary motor cortex, PM : premotor cortex, SMA: supplementary motor area, ACC: anterior cingulate cortex, PS: patients with psychomotor slowing, non-PS: patients without psychomotor slowing, SRRS: salpetriere retardation rating scale, M1_Inf_IE: inferior intereffector, M1_Mid_IE: middle intereffector, M1_Sup_IE: superior intereffector.

SI Appendix F: Complementary Methods

SI Appendix F1: Extended Methods sections

1. Participants

We combined data from two double-blind randomized placebo-controlled clinical trials, i.e., OCoPS-P (Overcoming Psychomotor Slowing in Psychosis; ClinicalTrials.gov Identifier: NCT03921450)[71] and BrAGG-SoS[75] (The Brain Stimulation And Group Therapy to Improve Gesture and Social Skills in Psychosis trial, NCT04106427)]. A substantial proportion of the MRI data from this sample was included in reports on grey matter structure in catatonia, structural brain correlates of formal thought disorder, cortical excitability in schizophrenia, as well as reliability of cerebral blood flow calculation and correlates of a finger tapping task[58, 76-78]. The sample included baseline data of 201 right-handed participants: 85 patients with schizophrenia spectrum disorders and psychomotor slowing according to the Salpêtrière Retardation Rating Scale[79] (Slowed group, SRRS total score ≥ 15) all from the OcoPS-P trial, 47 patients with schizophrenia spectrum disorders without psychomotor slowing (non-slowed group, SRRS score < 15) 23 from OcoPS-P and 24 from BrAGG-SoS, and 67 age and gender-matched healthy controls (HC group) 40 from OcoPS-P and 27 from BrAGG-SoS. Following the MRI preprocessing steps, two patients in the slowed group, four in the non-slowed group, and four participants in the healthy controls group were excluded due to poor neuroimaging data, leading to a final sample size of n = 189 (slowed group: n = 83, non-slowed group: n = 43, healthy controls: n = 63) (Table 1). The studies adhered to the Declaration of Helsinki and were approved by the local ethics committee (Kantonale Ethikkommission Bern, Switzerland: 2018-02164 and 2019-00798). Patient recruitment took place at the in- and out-patient departments of the University Hospital of Psychiatry and Psychotherapy, Bern, Switzerland. Healthy controls were recruited from the general population using flyers, online announcements, and word of mouth. Participants received written and oral information on the studies and had sufficient time to make their decisions. We ensured that the participants comprehended the information and received clarification in case of questions. Before any study procedures, participants provided written informed consent. Among the entire sample, eight patients were not on antipsychotic medication at the time of assessment. Daily dosage of antipsychotics was calculated as mean olanzapine (OLZ) equivalents[80].

The participants from the two trials did not differ in age, current medication dosage, number of episodes, duration of illness, as well as in the expert ratings and CR. However, patients from the OCoPS-P sample had higher PANSS ratings than those from the BrAGG-SoS trial (SI Appendix E1).

General exclusion criteria were active substance dependence except for nicotine, neurological disorders impacting motor behavior, severe brain injury with consecutive loss of consciousness, and contraindications for MR acquisition, i.e. metallic parts in the body, or pregnancy. Additional exclusion criteria for healthy controls were a history of any psychiatric disorder or any first-degree relative with psychosis.

2. Clinical and behavioral assessment

Patients were diagnosed with schizophrenia spectrum disorders (schizophrenia, schizoaffective, or schizophreniform disorders) according to the Structured Clinical Interview for DSM-5-TR (SCID-5). Symptom severity was assessed using the Positive And Negative Syndrome Scale (PANSS)[81]. Clinical rating scales of motor abnormalities included the Salpêtrière Retardation Rating Scale (SRRS[79]), the Bush Francis Catatonia Rating Scale (BFCRS)[82], and the Unified Parkinson's Disease Rating Scale Part III (UPDRS)[83]. Well-trained psychiatry residents conducted clinical ratings. Total gross motor activity was acquired using a tri-axial-accelerometer Move4 (movisens GmbH, Karlsruhe, Germany) strapped to the non-dominant arm for 24 hours. We calculated activity levels in counts/h during wake periods of the day[84-86]. Manual dexterity of the dominant hand was assessed using the coin rotation task (CR). During three trials, participants rotated a Swiss 50-Rappen coin, comparable to the size of a US dime with a diameter of 18.2mm, between thumb, index, and middle finger as fast as possible for 10 seconds. We calculated the CR score for each trial using the following validated formula: CR score=half turns−[(coin drops×.10)×half turns][87, 88],[73] and averaged the second and third trials.

3. MRI acquisition and preprocessing

We acquired a structural T1-weighted MRI scan and BOLD resting-state fMRI (rsfMRI) at the translational imaging center of the Swiss Institute for Translational and Entrepreneurial Medicine (SITEM), Bern, Switzerland. The MRI scans were acquired on a 3 T Prisma MRI whole-body scanner using a 20-channel radio-frequency head coil (Siemens, Germany). Patients lay horizontally in the MR scanner and their arms rested beside their trunk. We placed head motion foam pads around the patients' heads and explicitly instructed them to avoid head motion.
The MRI protocol included:

1- A structural scan using a T1-weighted MP2RAGE scan (8 minutes 22 seconds covering 176 sagittal slices, 1 mm thick, TR = 5000 ms, TE = 2.98 ms, flip angle 1= 4°, flip angle 2= 5°, voxel size = 1x1x1 mm).
2- A BOLD rs-fMRI scan using a multi-band echoplanar 2D (10 minutes and 11 seconds covering 600 volumes of 72 slices; 2.5 mm thick, TR =1000 ms, TE = 37 ms, flip angle =30 °, voxel size = 2.5×2.5×2.5 mm FOV = 230x230 mm, GRAPPA =1, multiband acceleration factor = 8).

Functional connectivity analyses were performed using CONN[89] release 22.a and SPM release 12.7771. We performed data preprocessing following the standard processing pipelines. We set the origin of all scans to the anterior commissure. Then, we performed motion realignment, co-registration between the functional scans and 3D-T1w scans of each participant, normalization to MNI space using DARTEL, and spatial smoothing with a Gaussian filter of 4 mm. Following these preprocessing steps, normalized functional data were denoised using a denoising pipeline similar to previous studies[90, 91] including the regression of potential confounding effects characterized by white matter, CSF, six motion parameters and their first order derivatives (12 factors), session effects and their first-order derivatives (2 factors), and linear trends (2

factors). This was followed by bandpass frequency filtering of the BOLD timeseries between 0.008 Hz and 0.09 Hz to exclude high-frequency fluctuations while minimizing the influence of physiological, head-motion, and other noise sources that might remain after the denoising step. We used an absolute head motion threshold involving translation greater than 2 mm or rotation greater than 2°, as well as a mean frame-wise displacement (FD) (as defined by Power et al.[(92)]) greater than 0.5mm as exclusion criteria.

4. MRI analyses

4.1 First-level analysis

To evaluate the resting-state functional connectivity (rs-FC) associated with the motor control of the dominant side, seven seeds (8 mm^3 spheres) were defined in the left hemisphere based on Gordon et al.[(23)] including the three intereffector (IE) areas (inferior, M1_Inf_IE (center -54 -3 14); Middle, M1_Mid_IE (center -38 -18 44), and Superior, M1_Sup_IE (center -19 -34 59), the three M1 effector areas (M1_Hand (center -35 -24 56), M1_Foot (center -4 -40 70), M1_Mouth (center -46 -15 34), and the SMA (center -5 -7 52) (see Figure 1A).

Seed-based rs-FC map for each of these seven seeds were estimated for each subject. Rs-FC strength was represented by Fisher-transformed bivariate correlation coefficients from a weighted general linear model (weighted-GLM), estimated separately for each seed area and target voxel, modeling the association between their BOLD signal time series.

4.2 Second-level analyses

First, we explored the seed-to-voxels rs-FC for seven seeds (the three intereffectors, the three M1 effectors (foot, hand, and mouth), and the SMA) within the whole sample. Particularly, we compared rs-FC patterns between adjacent seeds, e.g., M1_Foot vs. M1_Sup_IE, M1_Hand vs. M1_Mid_IE, and M1_Mouth vs. M1_Inf_IE).

Second, we compared seed-to-voxels rs-FC for the seven seeds between the three groups (healthy controls, patients with psychomotor slowing, and patients without slowing.

Third, we tested the association of seed-to-voxels rs-FC with objective measures of motor behavior (activity level from actigraphy, in a subset of patients with psychomotor slowing, n=76, healthy controls, n=40, patients without slowing, n=23, and manual dexterity from CR) in each of the three groups. Moreover, we tested seed-to-voxels rs-FC correlation with expert ratings of slowing (SRRS) within the slowed group. For the expert rating, we repeated the correlation analyses with the six seeds in the right hemisphere (M1_Inf_IE (54 -3 14); M1_Mid_IE (38 -18 44), M1_Sup_IE (19 -34 59), M1_Hand (35 -24 56), M1_Foot (4 -40 70) and M1_Mouth (46 -15 34)),

For all the analyses, we included the mean FD, age, and sex as covariates. In addition, analyses performed within and between patient groups included OLZ and PANSS total score as additional covariates to rule out the effect of daily medication and total symptom severity including negative symptoms.

4.3 Cross-validation analyses

To assess the robustness of seed-to-voxels rs-FC for the seven seed regions—the three intereffector regions, the three primary motor (M1) effectors (foot, hand, and mouth), and the supplementary motor area (SMA)—we employed a leave-out cross-validation approach. Ten subsets (n = 179 each) were generated by excluding 10 random participants from the full sample (n = 189). For each seed, seed-based connectivity masks were computed within each subset and compared to the corresponding connectivity mask derived from the full sample using the Dice Similarity Coefficient (DSC). We conducted an additional cross-validation procedure for the seeds that demonstrated rs-FC differences between patient groups (i.e., the middle and superior intereffector regions). Specifically, twenty subsets were created by excluding two random patients from each, and DSC values were computed.

The DSC is a measure for the spatial overlap of clusters and is defined as:

$$DSC = \frac{2 * |X \cap Y|}{|X| + |Y|}$$

where for each seed, X is the extent of the rs-FC of the full sample, and Y is the extent of rs-FC for each subset.

4.4 Statistics

A separate GLM was estimated for each seed (seed-to-voxels rs-FC). Voxel and cluster-level hypotheses were evaluated using multivariate parametric statistics with random-effects across subjects and sample covariance estimation across multiple measurements. The comparison of the connectivity between adjacent seeds represents within-subject effects, while the second-level analyses correspond to repeated-measures analyses of the selected effects (between seed comparisons within the same sample).

For seed-to-voxels rs-FC, cluster-level inferences were based on parametric statistics from Gaussian Random Field theory[93, 94] and the maps were thresholded using a combination of a cluster-forming $p<0.001$ (for the whole sample rs-FC) or $p<0.005$ (for the between-groups comparison) voxel-level threshold, and, to correct for multiple comparisons, a p-FDR(false discovery rate corrected) < 0.05 cluster-size threshold [95] [96]. We performed the cross-validation analyses at the same thresholds.

SI Appendix F2: Between-cohorts clinical comparisons.

Groups	OCoPS-P	BrAGG-SoS		
HC (n)	40	23		
PS (n)	83	-		
Non-PS (n)	22	21		
**Comaprison of non-PS**	**Mean ± SD**	**Mean ± SD**	**F-value**	**p-value**
**Age (years)**	35.6 **± 11.9**	**38.8 ± 12.3**	0.399	0.531
**OLZ equivalents (mg/d)**	**15.6 ± 10.7**	**13.2 ± 10.3**	1.102	0.3
**Episodes (n)**	**4.9 ± 6.6**	**5.9 ± 4.5**	0.091	0.764
**Duration of illness (years)**	**8.2 ± 7.7**	**14.5 ± 12.2**	0.085	0.798
**PANSS Total**	64.1±17.7	51.8±13.3	8.235	0.006
**PANSS Negative**	**15.3 ± 4.3**	**13.4 ± 4.8**	0.966	0.331
**PANSS Positive**	15.7±4.3	11.5±4.1	10.89	0.002
**SRRS**	**8.4 ± 2.9**	**8.8 ± 3.3**	0.3	0.587
**BFCRS**	**1.1 ± 0.9**	**2.3 ± 3.3**	3.905	0.055
**UPDRS III**	**9.6 ± 6.4**	**4.3 ± 4.1**	0.28	0.599
**CR**	**12.1 ± 3.1**	**12.8 ± 3.4**	0.408	0.527

Note: Olanzapine equivalents (OLZ), Positive And Negative Syndrome Scale (PANSS), Salpêtrière Retardation Rating Scale (SRRS), Bush Francis Catatonia Rating Scale (BFCRS), Unified Parkinson's Disease Rating Scale Part III (UPDRS), CR: coin rotation, HC: healthy controls, PS: patients with psychomotor slowing, non-PS: patients without psychomotor slowing. L: left, R: right.
